# Org 214007-0: A Novel Non-Steroidal Selective Glucocorticoid Receptor Modulator with Full Anti-Inflammatory Properties and Improved Therapeutic Index

**DOI:** 10.1371/journal.pone.0048385

**Published:** 2012-11-12

**Authors:** Marie-José C. van Lierop, Wynand Alkema, Anke J. Laskewitz, Rein Dijkema, Hans M. van der Maaden, Martin J. Smit, Ralf Plate, Paolo G. M. Conti, Christan G. J. M. Jans, C. Marco Timmers, Constant A. A. van Boeckel, Scott J. Lusher, Ross McGuire, Rene C. van Schaik, Jacob de Vlieg, Ruben L. Smeets, Claudia L. Hofstra, Annemieke M. H. Boots, Marcel van Duin, Benno A. Ingelse, Willem G. E. J. Schoonen, Aldo Grefhorst, Theo H. van Dijk, Folkert Kuipers, Wim H. A. Dokter

**Affiliations:** 1 Department of Immune Therapeutics, MSD, Oss, The Netherlands; 2 Department of Molecular Design and Informatics, MSD, Oss, The Netherlands; 3 Department of Pediatrics, Center for Liver Digestive and Metabolic Diseases, University Medical Center Groningen, Groningen, The Netherlands; 4 Molecular Pharmacology Department, MSD, Oss, The Netherlands; 5 Medicinal Chemistry Department, MSD, Oss, The Netherlands; 6 Women's Health Department, MSD, Oss, The Netherlands; 7 Department of Rheumatology and Clinical Immunology, University Medical Center Groningen, Groningen, The Netherlands; 8 Toxicology and Drug Disposition, MSD, Oss, The Netherlands; 9 Department of Laboratory Medicine, University Medical Center Groningen, Groningen, The Netherlands; University of Michigan Medical School, United States of America

## Abstract

Glucocorticoids (GCs) such as prednisolone are potent immunosuppressive drugs but suffer from severe adverse effects, including the induction of insulin resistance. Therefore, development of so-called Selective Glucocorticoid Receptor Modulators (SGRM) is highly desirable. Here we describe a non-steroidal Glucocorticoid Receptor (GR)-selective compound (Org 214007-0) with a binding affinity to GR similar to that of prednisolone. Structural modelling of the GR-Org 214007-0 binding site shows disturbance of the loop between helix 11 and helix 12 of GR, confirmed by partial recruitment of the TIF2-3 peptide. Using various cell lines and primary human cells, we show here that Org 214007-0 acts as a partial GC agonist, since it repressed inflammatory genes and was less effective in induction of metabolic genes. More importantly, *in vivo* studies in mice indicated that Org 214007-0 retained full efficacy in acute inflammation models as well as in a chronic collagen-induced arthritis (CIA) model. Gene expression profiling of muscle tissue derived from arthritic mice showed a partial activity of Org 214007-0 at an equi-efficacious dosage of prednisolone, with an increased ratio in repression versus induction of genes. Finally, in mice Org 214007-0 did not induce elevated fasting glucose nor the shift in glucose/glycogen balance in the liver seen with an equi-efficacious dose of prednisolone. All together, our data demonstrate that Org 214007-0 is a novel SGRMs with an improved therapeutic index compared to prednisolone. This class of SGRMs can contribute to effective anti-inflammatory therapy with a lower risk for metabolic side effects.

## Introduction

Synthetic glucocorticoids (GCs), like prednisolone, are among the most prescribed anti-inflammatory drugs for diseases like rheumatoid arthritis, inflammatory bowel disease and asthma. However, prolonged and/or high dosage GC treatment is associated with severe side effects such as insulin resistance, osteoporosis, skeletal muscle wasting, mood disorders and many others [1], [2]. The wide range of side effects is not surprising giving the essential role of the natural glucocorticoid cortisol in survival. For example, cortisol has a major role in blood glucose homeostasis and is essential for induction of stress-coping mechanisms. Effects of GCs are mediated via the ubiquitously expressed glucocorticoid receptor (GR), a member of the nuclear receptor super family. The small lipophilic GC molecule diffuses across the plasma membrane and binds to cytoplasmic GR that, upon binding of its ligand, alters its conformation and translocates to the nucleus. Within the nucleus, ligand-bound GR acts as a transcription factor that regulates expression of a set of target genes directly or indirectly. Direct regulation occurs via binding of activated GR homodimers to GC-response elements (GRE) in promoter regions of genes, thereby suppressing or inducing gene expression, the latter often termed “transactivation” (TA). Indirect regulation occurs by binding of activated GR to other transcription factors, hence facilitating or suppressing the action of these transcription factors, the latter often referred to as “transrepression” (TR) [3]. It has long been thought that the anti-inflammatory activity of GCs was mainly linked to TR activity, via interference with the two most important pro-inflammatory transcription factors, *i.e*., NFκB and AP-1 [4], [5]. The induction of metabolic side effects by GCs was thought to be mainly related to TA activity via the induction of many genes encoding enzymes that are active in metabolic pathways like glucose-6-phosphatase (G6Pase) and phosphoenolpyruvate carboxykinase (PEPCK). Because of these two clearly separated GC mechanisms of action it has long been hypothesized that it should be possible to design a GC molecule with preserved TR actions and reduced TA effects, resulting in an improved therapeutic index [6], [7], [8], [9]. Selection of novel selective GR modulators (SGRMs) with dissociating TA from TR activities *in vitro*, have so far only resulted in a few compounds with improved therapeutic profiles in animal models [6], [10], [11], [12]. To date, proof of concept in man remains to be obtained.

Due to a better understanding of the molecular interactions between the ligand-bound GR and its target molecules, awareness is growing that the concept of dissociation, based solely on TA vs TR, is too simplistic. For example, the anti-inflammatory activity of GCs is also partly driven by TA [13], [14], [15], [3], [7] In addition, results from ongoing studies in GRdim mice showed that some TA activity of GC is still required for the immune suppressive effect in a mouse model of RA [16]. Furthermore, besides interference with NFκB and AP-1, GC-bound GR also interacts with other transcription factors involved in immune responses like IRF-3, NFAT, STATs, GATA-3, T-bet and CREB [11], [17]. Of note, a recent publication indicates that a large number of genes involved in GC-induced side effects is regulated via a functional negative GRE in their promoter region [15]. Furthermore, a series of studies has shown that even subtle changes in the GR-ligand may alter conformation of the ligand-receptor complex with consequences for co-factor recruitment and hence for function of the receptor [9], [18], [19], [20], [21], [22], [23], [24].

Here we describe a novel non-steroidal, low-molecular weight GR ligand, Org 214007-0, the design of which was based on interference with the loop between helix 11 and helix 12 at the ligand-GR binding site. In comparison to prednisolone Org 214007-0 shows an overall improved therapeutic index in vitro as well as in vivo. Most importantly Org 214007-0 displays sustained full anti-inflammatory activity *in vivo* in mice, without adverse effects on plasma glucose levels and hepatic glucose metabolism.

## Results

### Design of Org 214007-0 and co-factor recruitment

**Figure 1:**
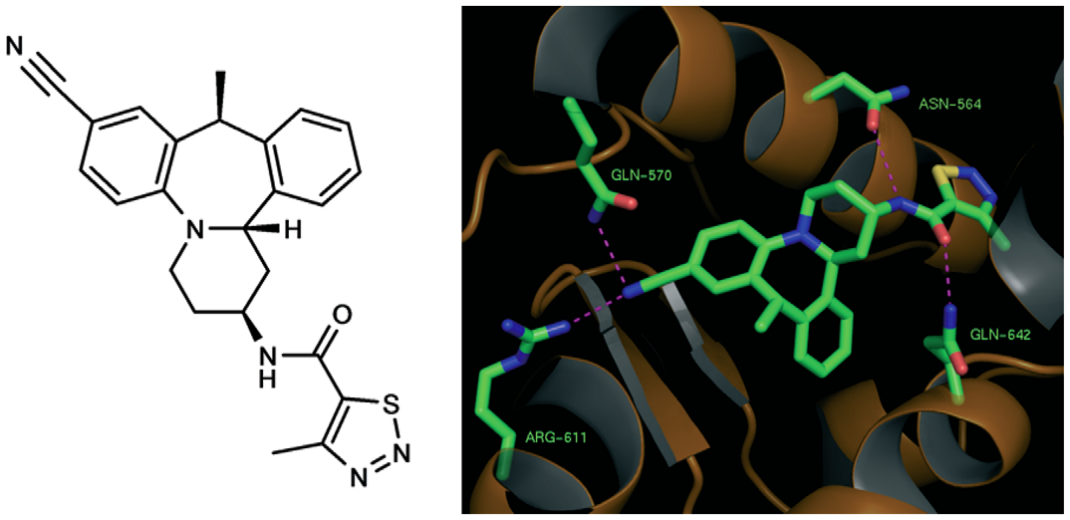
Structure and predicted binding mode of Org 214007-0. A) The structure of ORG 214007-0. This compound, [(-)-N-(2S,10S,14bS)]-N-(8-cyano-1,2,3,4,10,14b-hexahydro-10-methyl dibenzo*[c,f]*pyrido[1,2-*a*]azepin-2-yl)-4-methyl-1,2,3-thiadiazole-5-carboxamide] has a molecular weight of 430 g/mole (C_24_H_23_N_5_OS) B) The predicted binding mode of Org 214007-0 modeled in complex with the glucocorticoid receptor and demonstrating conservation of interactions typical to steroidal glucocorticoids (Gln564, Asn570, Arg611 and Gln642).

Org 214007-0, [(-)-N-(2S,10S,14bS)]-N-(8-cyano-1,2,3,4,10,14b-hexahydro-10-methyl dibenzo*[c,f]*pyrido[1,2-*a*]azepin-2-yl)-4-methyl-1,2,3-thiadiazole-5-carboxamide], the structure of which is shown in Figure 1A, has a molecular weight of 430 g/mole (C_24_H_23_N_5_OS). Synthesis of Org 214007-0 (described in detail in reference) [25] was based on both structural rationale and optimization of this class of tetracyclic compounds via molecular profiling, as will be described below. The structural rationale was based on GR mutation studies and ligand-based structure-activity relationships reviewed by Lusher et al [26]. These studies indicate that the loop between helix-11 and helix-12 of GR represents a hotspot that is crucial to the agonism/antagonism balance in GR, amongst others through modification of co-activator binding. In particular, modified binding of the third LXXLL motif in co-activators as Steroid Receptor Co-activator (SRC)-1 and Transcriptional Intermediary Factor (TIF)2 by conformational changes of this loop has been desribed [20], [22].

**Figure 2:**
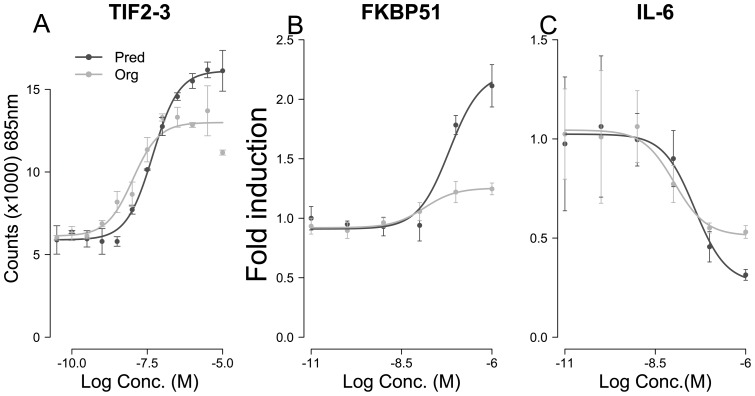
Org 214007-0 behaves as a partial agonist in vitro. A) In a co-factor recruitment assay, Org 214007-0, in comparison to prednisolone, shows potent but partial recruitment of a 0.1 μM peptide presenting TIF2 -3. On the Y-axis average fluorescence counts (+/− SD) are shown. EC50 values (%CV) and percentages maximal efficacy (%CV) for Org 214007-0 versus prednisolone were 10 (7.4) nM versus 48 (3.2) nM and 67 (7.1) % vs 100% respectively. B) In THP1 cells Org 214007-0 shows partial induction of FKBP51 protein expression and C) under inflammatory conditions represses the IL-6 protein expression almost as good as prednisolone does.

The strategy in the design of Org 214007-0 was therefore to interfere with this hotspot region, potentially inducing a conformational change of GR that differs from that induced by prednisolone and modifying co-activator binding. Figure 1B shows the predicted binding mode of Org 214007-0 modeled in complex with GR and demonstrates conservation of interactions typical to steroidal glucocorticoids (Asn564, Gln570, Arg611 and Gln642). To investigate whether Org 214007-0 in comparison to prednisolone indeed caused diminished recruitment of co-activator third motif LXXLL, a full dose-range of prednisolone and Org 214007-0 on recruitment of a peptide presenting TIF2 -3 was performed. The results showed that Org 214007-0, in comparison to prednisolone, recruited the peptide as a partial agonist, i.e. with similar potency but with lower efficacy (Figure 2A).

### In vitro profiling

Org 214007-0 was found to bind to GR as efficient as prednisolone (Ki of 2.2 nM *vs*. 3.8 nM for prednisolone) (Table 1, row A). For a full molecular profiling of Org 214007-0 and establishing whether changes in the Org 214007-0-induced GR conformation would specifically affect the expression of a selection of GR targetgenes in vitro, the following studies were performed in which Org 214007-0 was compared to prednisolone.

**Table 1:** Summary of *in vitro* studies.

	Cells/Assay	Stimulus	Read-out	Parameter	Unit	Pred	Org
**A**	**GR-binding**	-	competitive binding	Ki	nM	3.8 (±0.8)	2.2 (±1.3)
**B**	**CHO – ind**	-	MMTV-luc	EC50	nM	26.2 (±7.5)	5.1 (±1.6)
				Max. eff.	%	100	32 (±8.1)
**C**	**HepG2 – ind**	-	micro-array	Max eff.	%	100	67
**D**	**U2OS – rep**	IFNγ/TNFα	MCP-1	IC50	nM	0.74 (±2.3)	0.36 (±0.19)
				Max. eff.	%	100 (±9.6)	81 (±16)

Prednisolone (Pred) and Org 214007-0 (Org) were tested in different *in vitro* studies. A) *GR binding*: binding to recombinant human glucocorticoid receptor (GR) assessed by a fluorescence polarization competitor binding assay. Ki  =  inhibition constant or concentration of compound in the competitive binding assay which would occupy 50% of GR if no ligand was present. B) *CHO – ind*: Induction of gene expression measured in CHO cells stably co-transfected with human GR and a MMTV promoter – luciferase construct. C) *HepG2 – ind*: Induction of gene expression measured by microarray analysis of mRNA isolated from HepG2 cells incubated with either 1 μM prednisolone or 1 μM Org 214007-0. D) *U2OS – rep*: Repression of gene expression in U2OS cells overexpressing human GR. INFγ/TNFα – MCP-1  =  IFNγ (100 ng/ml)/TNFα (50 ng/ml) induced MCP-1 release.

IC50 or EC50 values represent the mean concentration of compound (±SD) required to resp. inhibit or effect the response to 50%. Maximal efficacy (Max. eff.) is expressed as the mean relative maximal effect (±SD) compared to the maximal effect by prednisolone (set at 100%). All assays (except for the micro array experiments) are performed at least two times.

GR-selectivity of Org 214007-0, either as agonist or antagonist, was evaluated by testing the compound in CHO cell lines, co-transfected with one of the human nuclear receptors and their respective reporter construct. In comparison to prednisolone, Org 214007-0 showed a strong potency but a clearly lower efficacy (32%, agonistic mode) in the GRE-mediated induction of gene expression in the human GR transfected cell line (Table 1, row B). No agonistic activity of Org 214007-0 for other human steroid receptors was detected. Org 214007-0 only possessed minor antagonistic activity for the human progestagen (B) and mineralocorticoid receptor, as shown in Table S1 in Text S1. In vivo activity of Org 214007-0 at therapeutic dose via PR was ruled out using the rabbit McPhail test, as shown in Table S2 in Text S1. To investigate whether in comparison to prednisolone the partial GR agonistic activity of Org 214007-0 would be sustained in cells that express endogenous GR and that are more relevant to the metabolic side effects of GCs, the human hepatocytic cell line HepG2 was used. Instead of focusing on a limited set of genes the genome-wide effect of both GCs was investigated by microarray analysis on total mRNA isolated from these cells The genes regulated by Org 214007-0 represented a subset of the genes regulated by prednisolone. However, the average maximal efficacy of induction of gene expression by Org 214007-0, compared to prednisolone (set at 100%) was 67% (Table 1, row C.) confirming partial agonistic activity of Org 214007-0 in these cells. For a few genes, i.e., tyrosine aminotransferase (TAT) and glucose 6-phosphatase (G6Pase) the partial induction by Org 214007-0 in comparison to prednisolone was confirmed for a whole dose-range of the compounds using Q-PCR (Figure S1). The anti-inflammatory activity of Org 214007-0 in comparison to prednisolone was quantified in a stably human GR transfected cell line (U2OS) stimulated to elicit an inflammatory response. Org 214007-0 was shown to inhibit this response with a potency comparable to that of prednisolone, while its efficacy was somewhat less compared to prednisolone (81%) (Table 1, row D).

**Figure 3:**
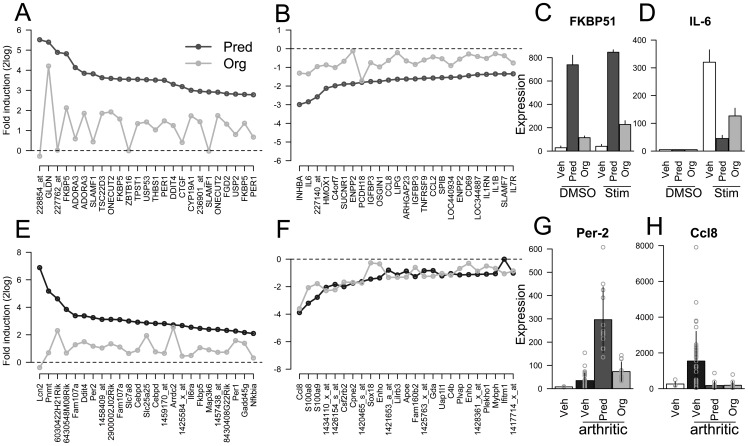
Org 214007-0 has a relatively lower impact on induction than on respression of genes. Fold changes for the top 25 genes either induced (A) or repressed (B) by 1 μM prednisolone and 1 μM Org 214007-0 in THP-1 cells. NB. Scales are in 2log. Example of an induced gene, FK506 binding protein 51 (FKBP51) (C) and a repressed gene, interleukin 6 (IL-6) (D) in comparison to the vehicle control under either non-stimulated or stimulated (IFNγ/TNFα) condition. Fold changes for the top 25 genes either induced (E) or repressed (F) by 1.5 mg/kg prednisolone and 0.3 mg/kg Org 214007-0 in muscle tissue from arthritic mice. NB. Scales are in 2log. Example of an induced gene (Per-2) (G) and a repressed gene (Ccl8) (H) in comparison to vehicle treated arthritic mice and vehicle treated healthy mice.

A key next phase in profiling Org 214007-0 against prednisolone was to compare both repression and induction of gene expression within the same cell, which allows for the determination of a relative therapeutic index (TI) defined as the ratio between % efficacy by which genes are repressed/% efficacy by which genes are induced in comparison to prednisolone (set at 1 = 100%/100%). Org 214007-0 and prednisolone were tested in the human monocytic THP1 cell line and microarray analyses were performed to assess both induction of gene expression and repression of gene expression under an inflammatory condition. All the genes that were either induced or repressed by Org 214007-0 were also regulated by prednisolone: yet, in all cases the fold-induction of genes by Org 214007-0 was less and the fold-repression of genes was equal to or less than that by prednisolone. Shown in Figure 3A and 3B are the gene sets ranked on basis of the magnitude of up- and down-regulation by prednisolone respectively. The top 25 genes from each set were used for further evaluation in which we calculated a mean fold change for either the induction or repression of genes by both compounds (see Figure S2). These calculations show that in this cell system Org 214007-0 shows an average maximal gene induction of 19.5% and an average maximal gene repression of 48.3% resulting in a 2.5 fold improvement of the TI relative to prednisolone (Table 2, row A). One gene that is induced (FKBP51; Figure 3C) and one gene that is repressed (IL-6; Figure 3D) are shown as examples. For a small selection of genes, these differences in expression levels were confirmed, comparing a whole dose range of Org 214007-0 and prednisolone by either Q-PCR for FKBP51, GILZ and DUSP (Figure S3) or by specific AlphaLISAs for FKBP51 (Figure 2B) IL-6 (Figure 2C), MCP-1, and IL-8 (Figure S3).

**Table 2:** Summary of studies to define the therapeutic index (TI) of Org 214007-0.

	Cells/Assay	Stim	Read	Param	Unit	Pred	Org	TI_rel_
**A**	**THP-1 – rep**	-	m.a.	Max. eff.	%	100	48.3	
	**– ind**	I/T	m.a.	Max. eff,	%	100	18.9	2.5
**B**	**THP-1 – rep**	I/T	MCP-1	IC50	nM	69.9 (±30.4)	23.5 (±9.3)	
				Max. eff.	%	100	44 (±8)	
			IL-6	IC50	nM	34.1 (±6.6)	9.3 (±2.1)	
				Max. eff.	%	100	78 (±9)	
			IL-8	IC50	nM	36.9 (±11.3)	11.0 (±3.1)	
				Max. eff.	%	100	74 (±10)	
**C**	**THP-1 – ind**	-	FKBP51	EC50	nM	65.8 (±22.4)	8.0 (±5.3)	2.0
				Max. eff.	%	100	42 (±8)	
			GILZ	EC50	nM	60.2 (±19.6)	13.7 (±11.8)	
				Max. eff.	%	100	13 (±5)	
			DUSP1	EC50	nM	67.4 (±8.4)	6.4 (±4.1)	
				Max. eff.	%	100	41 (±6)	
**D**	**hWB – rep**	LPS	TNFα	IC50	nM	27 (±5.7)	50 (±19)	
				Max. eff.	%	100	68 (±8.7)	
		P/28	IL-5	IC50	nM	6.76 (±1.5)	14.45 (±3.2)	
				Max. eff.	%	100	64 (±11)	
	**– ind**	P/28	G-CSF	EC50	nM	67.61 (±3.8)	186.2 (±63)	1.7
				Max. eff.	%	100	39 (±7.8)	
**E**	**CASM3C – rep**	1/I/T	MCP-1	Max. eff.	%	100	98	
	**– ind**	-	SAA	Max. eff.	%	100	58	1.7
**F**	**HDF3CGF – rep**	1/I/T	MMP-1	Max. eff.	%	100	120	
	**– ind**	-	PAI-1	Max. eff.	%	100	55	2.2
**G**	**CIA mus. – rep**		m.a.	Max. eff.	%	100	87.7	
	**– ind**		m.a.	Max. eff.	%	100	25.7	3.4

A) *THP-1*: Microarray (*m.a*.) analysis of mRNA isolated from THP-1 cells incubated for 6 hours with either 1 μM prednisolone or 1 μM Org 214007-0 without (*ind*) or with IFNγ (220 ng/ml)/TNFα (374 ng/ml) ( = *I/T*), (*rep*) The mean percentage repression or induction of genes compared to that by prednisolone (set at 100%) is indicated. B) *THP-1 – rep:* Repression of gene expression in THP-1 cells. *I/T – MCP-1, IL-6, IL-8*  =  TNFα (60 ng/ml)/IFNγ (40 ng/ml) induced MCP-1, IL-6 or IL-8 release. C) *THP-1 – ind*: Induction of FK506 binding protein 51 (*FKBP51*), glucocorticoid induced leucine zipper (*GILZ*) and dual specificity phosphatase 1 (*DUSP1*) in THP-1 cells. D) *hWB* – *rep:* Inhibition of LPS-induced (*LPS*) TNFα release or PMA/anti-CD28 (*P/28*) induced IL-5 release by primary human whole blood cells and *hWB – ind*: enhancement of PMA/anti-CD28/compound (*P/28*) induced G-CSF release by primary human whole blood cells. E) *CASM3C – rep*: Inhibition of MCP-1 release of coronary artery smooth muscle cells ( = *CASM3C*) stimulated with a cytokine mixture of IL-1β (1 ng/ml), IFNγ (100 ng/ml) and TFNα (5 ng/ml) ( = *1/I/T*) by 1 μM prednisolone or 1 μM Org 214007-0. *CASM3C – ind*: Induction of serum amyloid A (*SAA*) of the cells mentioned above by 1 μM prednisolone or 1 M Org 214007-0. F) *HDF3CGF – rep*: inhibition of matrix metalloproteinase (*MMP-1*) release of human neonatal foreskin fibroblasts stimulated with the cytokine mixture mentioned above plus required growth factors ( = *HDF3CGF*). *HDF3CGF – ind*: Activation of plasminogen activator inhibitor-1 (*PAI-1*) by cells mentioned above by 1 μM prednisolone or 1 μM Org 214007-0. G) *CIA mus*.: Microarray (*m.a*.) analysis of mRNA isolated from muscle cells isolated at day 21, 2.5 hours after the final oral administration of either 1.5 mg/kg prednisolone or 0.3 mg/kg Org 214007-0 in the mouse CIA experiment. The mean percentage repression (*rep*) or induction (*ind*) of genes compared to that by prednisolone (set at 100%) is indicated.

IC50 or EC50 values represent the mean concentration of compound (±SD) required to resp. inhibit or effect the response to 50%. Maximal efficacy (Max. eff.) is expressed as the mean relative maximal effect (±SD) compared to the maximal effect by prednisolone (set at 100%). A relative therapeutic index (TI_rel_) is calculated by the ratio of (the mean) % maximal efficacy in repression and (the mean) % maximal efficacy in induction of genes by Org 214007, where that of prednisolone is set at 1 (100%/100%). All assays (except for the microarray experiments) are performed at least two times.

**Figure 4:**
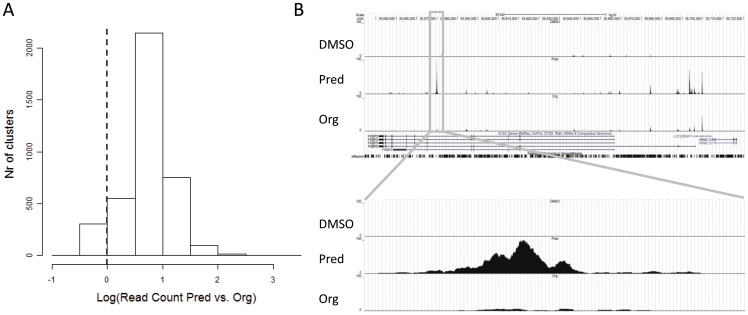
Org 214007-0 leads to relatively less GR occupancy. A) Ratio of the read count for clusters induced by 1 μM prednisolone or 1 μM Org 214007-0 in a ChIP-Seq analysis. If Org 214007-0 and prednisolone would induce an equal number of reads, the histogram would be centered on Log0, indicated by the dotted line. There is a clear shift to the right from this line (P-value (mean  = 0) <0.000001), indicating that prednisolone leads to more GR occupancy than Org 214007-0. B) Example profile of the tag clusters in the GR response gene FKBP51. Multiple binding sites are found within this gene, each showing denser clusters after treatment with 1 μM prednisolone than after treatment with 1 μM Org 214007-0. The inset highlights an intronic region at 87 kb downstream of the transcription start site. This region that was identified by Paakinaho et al. (2010) [54], as a major intronic enhancer in human A459 lung cancer cells and was shown to be occupied by GR after treatment with the GR ligand dexamethasone.

In order to define in more detail the molecular mechanism that underlies the differential effects of Org 214007-0 compared to prednisolone on gene expression, we performed ChIP-Seq analysis on DNA from the THP-1 cells. Compared to vehicle, the numbers of clusters found for Org 214007-0 versus prednisolone under the non-stimulated condition were 2080 *vs*. 9413, respectively. An example of these clusters in the FKBP51 gene is shown in Figure 4B. Under the inflammatory condition, the numbers of clusters were 2502 *vs*. 3471. So, under both conditions, Org 214007-0, causes less binding of GR to the DNA than prednisolone does. Comparison of the set of 2080 putative binding sites of Org 214007-0 with the 9413 binding sites of prednisolone showed that 99.5% of the binding sites in the Org 214007-0 set were also contained within the prednisolone set, indicating that Org 214007-0 is a genuine GC that does not lead to binding of GR on sites that are not targeted by prednisolone. This is in close agreement with the microarray data in which a similar large overlap was shown between the 2 gene sets. The ratio of the read count for clusters induced by prednisolone or Org 214007-0 is shown in Figure 4A as a histogram. Clearly, most clusters have a ratio greater than 1 (2log ratio larger than 0), indicating that prednisolone leads to more GR occupancy than Org 214007-0 does.

Next, effects of Org 214007-0 on the cytokine release by primary human whole blood cells treated with either LPS or anti-CD28/PMA were tested. As depicted in Table 2, row D, maximal efficacies of Org 214007-0 again were partial compared to prednisolone, resulting in a relative TI of 1.7 (additional data are shown in Table S3 in Text S1). Potencies of both compounds were in the same order of magnitude (see IC50's in Table 2, row D) In addition, Org 214007-0 and prednisolone were compared in some of the Biologically Multiplexed Activity Profiling (BioMAP) assays at BioSeek, Inc. (Burlingame, CA, USA). These assays make use of different types of primary human cells, in this case coronary artery smooth muscle cells and neonatal foreskin fibroblasts, in an inflammatory environment [27], [28]. As can be seen in Table 2, rows E and F, Org 214007-0 showed full efficacies in the two cell systems, i.e., 98% and 120% repression of MCP-1 and MMP-1, respectively. Also in these cells, partial induction of protein expression by Org 214007-0 in comparison to prednisolone was observed, i.e. 58% for serum amyloid A and 55% for plasminogen activator inhibitor-1. When all repressed and induced proteins are taken into account, a relative TI value for Org 214007-0 could be deduced of 1.7 in coronary artery smooth muscle cells and of 2.2 in neonatal foreskin fiboblasts.

### *In vivo* studies

**Figure 5:**
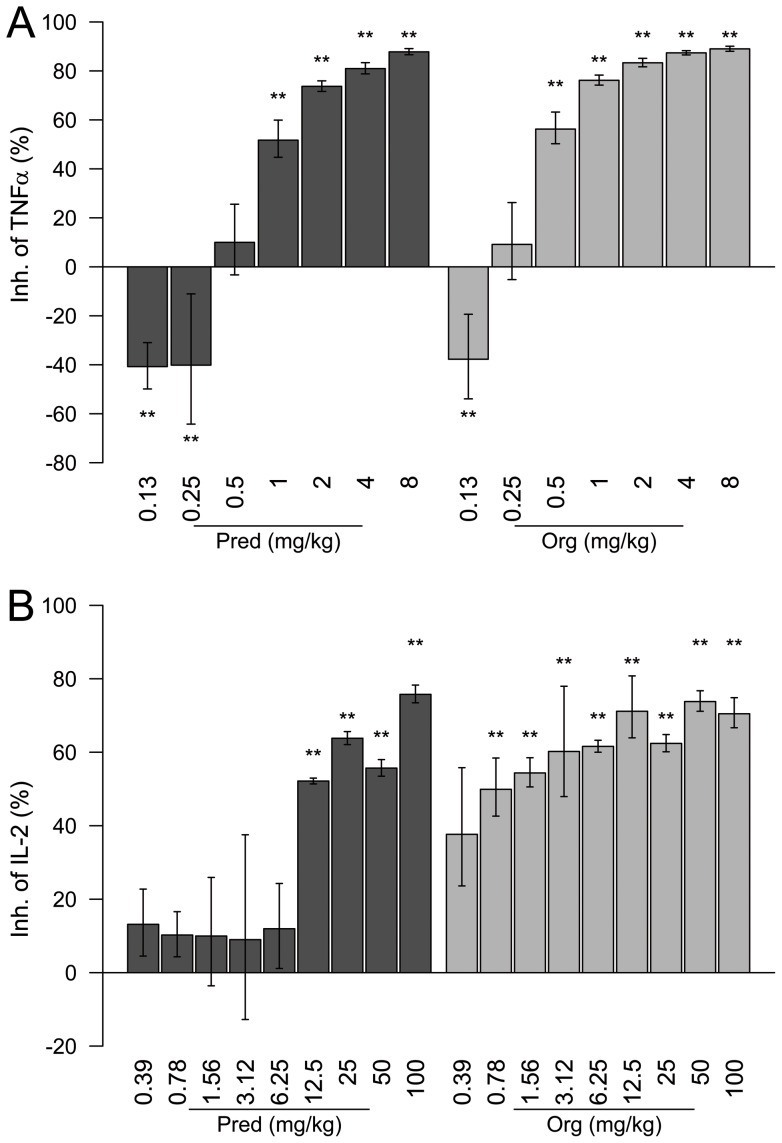
Org 214007-0 is equally effective as prednisolone in acute inflammation mouse models. A) Inhibition of acute inflammation in the LPS-induced TNFα mouse model. Mean percentage inhibition (of each group of n = 8) (± SEM) of the LPS-induced TNFα release by the vehicle treated group is shown (no TNFα is detectable without LPS injection). **  =  significantly different from placebo (p<0.01; ANOVA-test). B) Inhibition of acute T cell-driven inflammation in the anti-CD3-induced IL-2 mouse model. Org 214007-0 shows a more potent activity than prednisolone in this model. Mean percentage inhibition (of each group of n = 2) (± SEM) of the anti-CD3-induced IL-2 release relative to a positive control group (treated with 10 mg/kg A420983, a pan-SRC inhibitor) (set at 100%) is shown (no IL-2 is detectable without anti-CD3 injection). **  =  significantly different from placebo (p<0.01; ANOVA-test).

To evaluate how the *in vitro* profile of Org 214007-0 would translate to the *in vivo* situation with regard to both anti-inflammatory efficacy and induction of side effects, the compound was tested head-to-head to prednisolone in several *in vivo* models. Since pharmacokinetic studies in mice showed good oral bio-availability of Org 214007-0 and prednisolone (shown in Table S4 in Text S1), both compounds were dosed orally in all preclinical mouse models. Firstly, Org 214007-0 was tested in an acute inflammation model for its potency to inhibit the LPS-induced raise in serum TNFα. As can be seen in Figure 5A, the potency of Org 214007-0 in this model was stronger than that of prednisolone (ED50 0.5 mg/kg *vs*. 1 mg/kg). Surprisingly, and in contrast to its *in vitro* repression efficacy on LPS-induced TNFα, the maximal efficacy of Org 214007-0 in this model was as good as that of prednisolone, reaching a 90% inhibition of the LPS-induced TNFα release. Administration of GR antagonist RU486 (mifepristone) to the mice just prior to oral treatment with either Org 214007-0 or prednisolone fully antagonized this inhibition (data for Org 214007-0 in Figure S4), confirming that inhibition of the TNFα response was indeed mediated via GR. The effect of Org 214007-0 was also tested in a T-cell-driven inflammation model using anti-CD3-induced IL-2 release as readout. As can be seen in Figure 5B, Org 214007-0 dose-dependently reduced the anti-CD3 induced IL-2 serum levels with a similar efficacy as prednisolone (75% inhibition) but with a stronger potency than prednisolone (ED50 approximately 0.3 mg/kg *vs*. ca. 10 mg/kg). This again demonstrates that, although Org 214007-0 behaves as a true partial GR agonist *in vitro*, it shows anti-inflammatory activity as good as prednisolone *in vivo*.

**Figure 6:**
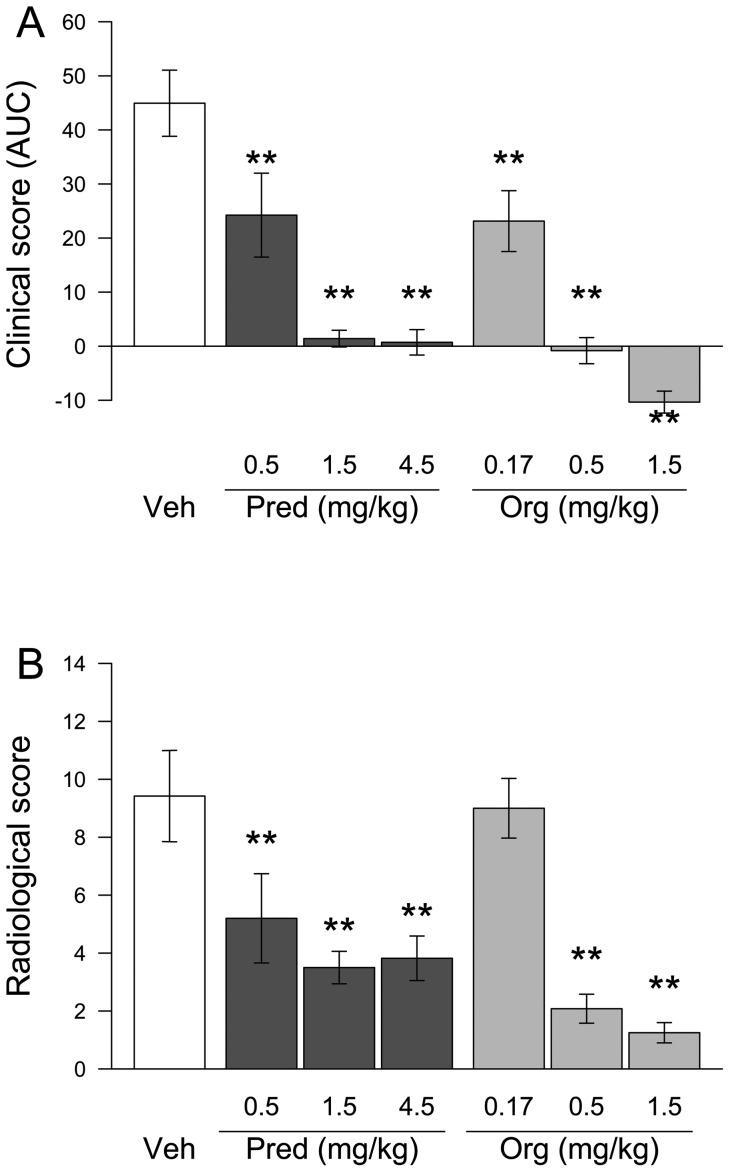
Org 214007-0 is equally effective as prednisolone in the mouse CIA model. A) Inhibition of arthritis in the CIA model. Mean clinical score of each group (n = 12), shown as area under the curve (AUC) of the arthritis score monitored every other day during 3 weeks, corrected for baseline, is indicated (± SEM). **  =  significantly different from placebo (p<0.01; ANOVA-test). B) Reduction of bone destruction in the CIA model as measured by X-ray. Mean radiological score (sum of the X-ray scores of left and right hindpaws and knees) of each group of mice (n = 12) at the end of the CIA experiment is indicated (± SEM). **  =  significantly different from placebo (p<0.01; ANOVA-test).

We next tested the efficacy of the compound in a more relevant chronic disease model in which the classical GCs have proven efficacy. The murine collagen-induced arthritis (CIA) model is a well-accepted model of human rheumatoid arthritis, encompassing inflammation of synovial joints, destruction of cartilage and bone erosion. Org 214007-0 and prednisolone were dosed orally (once daily 3 weeks) in a therapeutic manner, i.e., treatment started when disease was established. Both prednisolone and Org 214007-0 caused a dose-dependent reduction of the disease score (Figure 6A). Org 214007-0 was found to be about 3-fold more potent than prednisolone in this model, leading to a total suppression of the disease symptoms at a dose of 1.5 mg/kg/day. Besides the clinical score of the paws, the two highest doses of Org 214007-0 (0.5 and 1.5 mg/kg/day) also showed a significant reduction in bone damage as determined by X-ray on knees and paws at the end of the study (Figure 6B), indicating a reduction of disease progression. Finally, histopathologic examination of the inflamed knee joints also showed a significant reduction of inflammatory infiltrates, cartilage destruction and bone apposition in mice treated with the two highest doses of Org 214007-0 (Figure S5). Altogether these results demonstrate that the anti-inflammatory *in vivo* efficacy of Org 214007-0 is as good as or even better than that of prednisolone.

In the same chronic disease model we wanted to test whether the partial activity of Org 214007-0 on induction of gene expression and its improved TI, as observed *in vitro*, would be sustained *in vivo*. Evaluation of the gene expression profiles induced by prednisolone in different tissues and at different time points after the last dosing of compound in a CIA experiment indicated that muscle tissue collected 2.5 hours after dosing provided representative and robust gene expression data. Therefore, muscle tissue was collected from arthritic mice that were treated for 3 weeks with either vehicle only or with dosages of Org 214007-0 and prednisolone that were equally effective in inhibiting arthritis. Besides these groups of mice, one group of healthy mice (sham-immunized and vehicle-treated) was included. Collected tissue was used to isolate mRNA for microarray analysis. The top 25 genes (represented by the probes on the chip) that were at least 2-fold up-regulated by either Org 214007-0 or prednisolone in comparison to vehicle treated mice are shown in Figure 3E Among these genes are, a.o., FoxO1 and bona fide GR target genes like Fkbp51 and Per-2 (the latter shown as an example in Figure 3G). Genes that were at least 2-fold up-regulated by disease induction (vehicle-treated arthritic *vs*. vehicle-treated healthy mice) and that were down-regulated at least 2-fold by either Org 214007-0 or prednisolone are shown in Figure 3F. Among the genes are some well-known inflammatory markers as S100A8, S100A9 and Ccl8, the latter shown as an example in Figure 3H. The fold induction of gene expression by Org 214007-0 was always lower than that caused by prednisolone, whereas fold repression of gene expression was more or less equal for Org 214007-0 and prednisolone. We also calculated a mean fold change for all induced and repressed genes by both compounds derived from this microarray. Based on these calculations Org 214007-0, relative to prednisolone, shows an average maximal gene induction of 25.7% and an average maximal gene repression of 87.7%. In other words, also *in vivo* Org 214007-0 shows a relatively low effect on induction of gene expression compared to prednisolone at dosages that are equally effective in suppression of arthritis leading to a relative TI of 3.4 (Table 2, row G).

**Figure 7:**
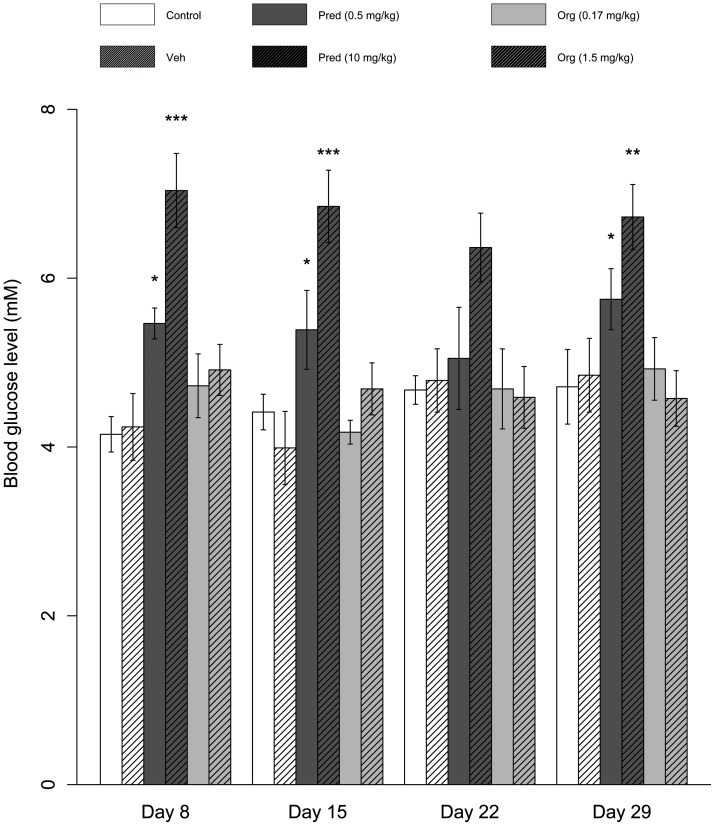
Org 214007-0 induces less elevated fasting glucose levels. Mice (n = 8 per group) are not treated (Control) or treated po, once daily, for 28 days with either vehicle only, prednisolone 0.5 mg/kg/day or 10 mg/kg/day or Org 214007-0 0.17 mg/kg/day or 1.5 mg/kg/day. Both the two lowest doses of each compound are equi-efficacious in suppression of CIA as well as the two highest doses. Blood glucose levels (mean ± SEM) were measured at day 8, 15, 22 and 29, after 9 hours of fasting. * p<0.05, ** p<0.01, *** p<0.001: significantly different (Student's t-test) from vehicle treated group.

Finally, we assessed whether Org 214007-0 indeed has a favorable TI with respect to its (side-) effects on glucose metabolism. Mice treated daily with prednisolone for 28 days showed, in comparison to vehicle treated mice, significantly elevated fasting blood glucose levels (p<0.01) from day 8 onwards. Interestingly, in mice treated with equipotent doses of Org 214007-0 fasting blood glucose levels were not affected (Figure 7).

**Figure 8:**
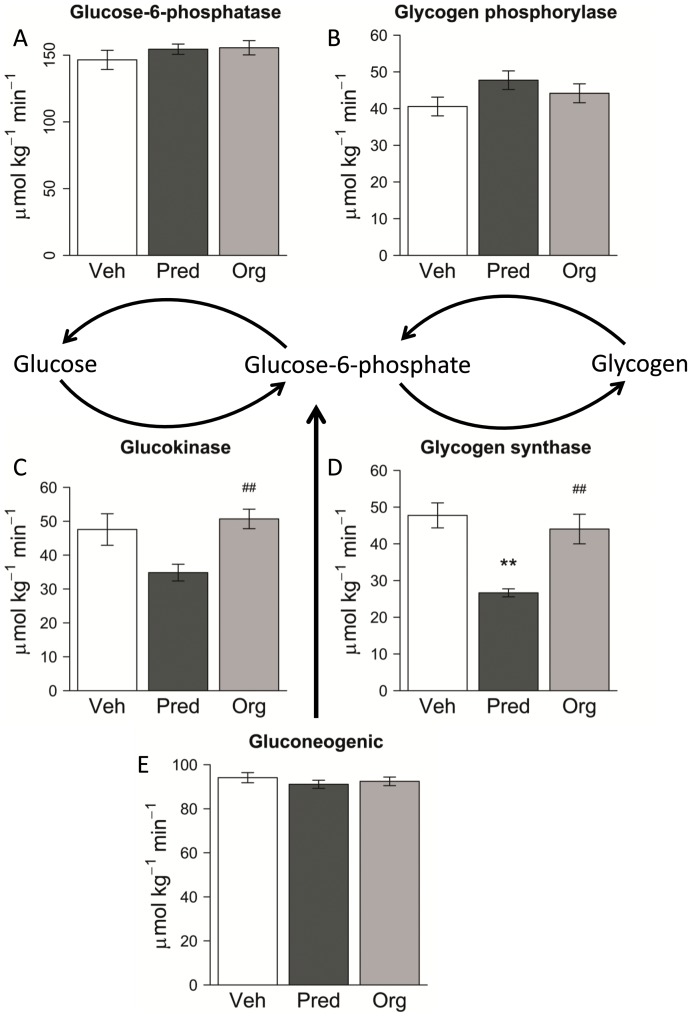
Org 214007-0 does not effect rates of hepatic enzyme fluxes. Mass Isotopomer Distribution Analysis (MIDA), as described in detail in Materials and Methods, was performed in mice treated p.o., once daily, for 7 days with either vehicle, prednisolone (10 mg/kg) or Org 214007-0 (1.5 mg/kg). These doses of each compound are equi-efficacious in suppression of CIA. Neither the glucose-6-phosphatase flux (A) nor the glycogen phosphorylase flux (B) were affected by treatment with prednisolone or Org 214007-0. The glucokinase flux rate (C) was not changed by Org 214007-0, but significantly differed from the effect by prednisolone (##: p = 0.01 *vs* prednisolone). The glycogen synthase flux rate (D) was significantly decreased by prednisolone (**: p = 0.005 *vs* vehicle), whereas Org 214007-0 had no significant effect on this flux, but differed significantly from prednisolone (##: p = 0.002 *vs* prednisolone). Neither Org 214007-0 nor prednisolone, at equi-efficacious dosages, effects the gluconeogenic flux (*de novo* synthesis of glucose-6-phophate) (E).

**Figure 9:**
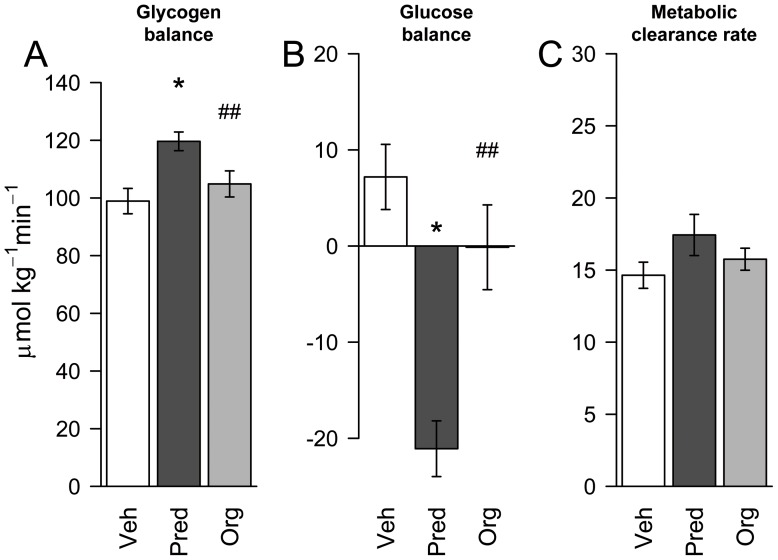
Org 214007-0 does not cause a shift in the liver glucose/glycogen balance. As determined in MIDA (details in legend Figure 8), the glucose balance (A), which represents the difference between the glucose-6-phophatase and glucokinase flux, is not affected by Org 214007-0 treatment but is significantly changed by the prednisolone treated group (*: p = 0.046 *vs* vehicle.; ##: p = 0.004 *vs* prednisolone). The glycogen balance (B), representing the difference between the glycogen synthase and glycogen phophorylase flux, is not effected by Org 214007-0 treatment but is significantly changed by the prednisolone treated group (*: p = 0.024 *vs* vehicle; ##: p = 0.005 *vs* prednisolone). Neither Org 214007-0 nor prednisolone, at equi-efficacious dosages, effects the metabolic clearance rate of blood glucose (C).

To evaluate the effects of the compound on hepatic glucose metabolism, a mass isotopomer distribution analysis (MIDA) approach was performed. Mice were dosed for 7 days with Org 214007-0 (1.5 mg/kg) or prednisolone (10 mg/kg) or vehicle only. Daily oral treatment with these dosages of Org 214007-0 and prednisolone result in more or less equal molar total exposure levels of both compounds and in equal inhibition of arthritis in the CIA model as shown previously. Also in this study, the fasting blood glucose levels in mice treated with prednisolone were significantly elevated compared to vehicle-treated mice (p<0.05), while Org 214007-0 did not affect fasting blood glucose level (Figure S6). The hepatic flux rates calculated from the MIDA results are shown in Figure 8. The glucokinase and glycogen synthase flux rates were significantly decreased by prednisolone whereas Org 214007-0 had no significant effects. These differences in flux rates resulted in differences in glucose and glycogen balances as shown in Figure 9. Since prednisolone treatment caused no change in the gluconeogenic flux (from pyruvate to glucose-6-phosphate) (Figure 8) nor in the blood glucose metabolic clearance rate (Figure 9), the effect of prednisolone on the glucokinase and glycogen synthase flux rates may explain the increase in fasting glucose levels in prednisolone-treated mice. Org 214007-0 did not alter these balances, confirming a significantly different mode of action of glucose metabolism in comparison to prednisolone.

Taken together, the *in vitro* partial GR agonistic profile of Org 214007-0 translates *in vivo* into a full anti-inflammatory compound with less adverse effects on glucose metabolism in mice. Org 214007-0 therefore represents a new class of SGRMs with an improved therapeutic index in comparison to prednisolone. This class of compounds is highly interesting for further development towards improved oral SGRMs for clinical use.

## Discussion

About two decades ago, the concept of a “dissociating glucocorticoid” was defined, based on the discovery of the two different mechanisms of glucocorticoids action: transrepression *vs* transactivation. This concept was enforced by the finding that DNA-binding of the GR-ligand complex was not required for transrepression [29], [5]. Since then, drug discovery programmes have been initiated on dissociating glucocorticoids (nowadays often referred to as SGRMs), resulting in the development of a number of compounds that indeed show improved TR/TA ratio *in vitro* (reviewed in reference) [11]. However, only a limited number of compounds also show a dissociating profile *in vivo* when tested in preclinical animal models. The hypothesis that an improved TR/TA ratio is beneficial in the clinic still awaits its proof of concept in man. In addition it is now evident that the simple concept of TR versus TA activity requires revision based on new insights in the complexity of the mechanisms of GC action. Some TA activity still seems to be required for the anti-inflammatory action of GCs [3], [7], [13], [14], [15], whereas some side effects of GCs are thought to be driven by transrepression [30]. An exception to this rule seems to be observed with the plant-derived compound CpdA that shows characteristics of a full dissociating GC, lacking any TA activity [31]. However, the mechanism of action of this compound seems to differ from a true GC agonist or antagonist, preventing GR-dimer formation and GR-DNA binding [32].

In this paper we provide an extended *in vitro* characterization combined with specific *in vivo* data on a SGRM that belongs to a new chemical class of non-steroidal low molecular weight compounds, i.e., Org 214007-0. Org 214007-0 binds with high affinity to GR, comparably to prednisolone. *In vitro*, Org 214007-0 behaved as a partial GR-selective agonist with a potency comparable to that of prednisolone. The maximal efficacy on induction of gene expression by Org 214007-0, in comparison to prednisolone, appeared to be partial. The maximal efficacy on repression of inflammatory responses by Org 214007-0 was also partial but more close to that of prednisolone, resulting in an improved relative therapeutic index (TI) for Org 214007-0 of about 2. The typical *in vitro* profile of Org 214007-0 was confirmed in several cell lines (HepG2, THP-1) for the total array of GC modulated genes. In addition, this unique profile of Org 214007-0 was confirmed in several types of human primary cells under different pro-inflammatory conditions.

Although Org 214007-0 binds GR with the same affinity as prednisolone, the conformation of the GR-Org 214007-0 complex leads to a lower binding affinity to the DNA GR-binding sites in comparison to the GR-prednisolone complex, as was shown by a ChIP-Seq study. These data are in agreement with the partial activity of Org 214007-0 on gene induction. Modeling the binding-mode of Org 214007-0 to GR suggests that the molecular basis for the compounds' partial agonism involves disturbance of the loop region between helix-11 and helix-12 rather than a direct clash with helix-12 itself. The importance of this loop region in mediating agonism and antagonism for GR has recently been reviewed [26]. Since co-modulator binding depends on the position of this helix, this will probably affect recruitment of co-activators and thereby gene induction activity [22], [33]. Recruitment of peptide TIF2-3, previously described to be affected by this region [20], [22] was indeed shown to be partial with Org 214007-0 bound GR.

The *in vivo* activity of Org 214007-0 was tested in several mouse models. Two acute inflammation models demonstrated the anti-inflammatory activity of Org 214007-0 at the level of both monocytic cells (LPS-induced TNFα model) and T cells (anti-CD3-induced IL-2 model). Strikingly, Org 214007-0 was found to behave as a potent and full GR agonistic anti-inflammatory agent in these models. Even more importantly, this strong potency and full anti-inflammatory efficacy was sustained in a chronic disease model, i.e., the CIA model. Microarray analysis on muscle RNA from these mice showed that the efficacy in suppression of the CIA disease score was in line with a reduction of disease-related or pro-inflammatory genes like TLR4 ligand S100A8/S100A9 [34] and monocyte chemotactive protein-2 (chemokine[C-C]-ligand 8 or CCL8) [35]. More importantly, the partial activity of Org 214007-0 on gene induction, as observed *in vitro*, was sustained in the muscle in the CIA model, since the GR-dependent induction of genes was always lower upon Org 214007-0 treatment compared to prednisolone. A large part of the induced genes are well-known GR-regulated genes like FKBP51, FoxO1, phenylethanolamine-N-methyltransferase (PNMT), Ddit4 (or REDD1), Lcn2 (or Lipocalin 2) and Per-2 [7], [36]. Interestingly, Fox01 was shown to play a role in muscle atrophy, one of the main unwanted side effects of chronic GC-treatment. However, Waddell et al [37] show that Fox01 effects on muscle are mediated through direct induction of MuRF1 expression, whose promoter contains both a GRE and a Fox01-binding element. Despite upregulation of Fox01, MuRF1 was not regulated in our study in muscle of either prednisolone or Org 214007-0-treated mice. Further studies are needed to clarify whether our present finding really represent a discrepancy with existing literature or whether the different experimental conditions like different kinetics of mRNA expression give rise to this apparent discrepancy. Moreover, it will be of great interest to do dedicated studies on muscle wasting to try to link the gene-expression profiles of Org 214007-0 and prednisolone to clinical consequences on muscle mass. Another interesting gene is Per-2, which belongs to the class of circadian clock genes and has recently been described as a primary GR target gene involved in glucose homeostasis [38]. It is tempting to speculate that a less pronounced induction of Per-2 by Org 214007-0 compared to an equi-efficacious dose of prednisolone contributes to a better metabolic side effect profile of Org 214007-0. However, a functional metabolic side effect profile could not be derived from our CIA model, since we have not seen any induction of either glucose or insulin at a dose of 1.5 mg/kg/day prednisolone in our CIA experiments. Other groups have described increased fasting glucose or insulin levels in mice treated with prednisolone in acute inflammation models [6], [39] and in a chronic disease model [40]. Riether et al. [40] showed that 30 mg/kg prednisolone induced significantly elevated serum insulin levels in a mouse CIA experiment. However, the dose of prednisolone used by Riether et al. was much higher than the dose (1.5 mg/kg/day) that is fully efficacious in our CIA model. To gain direct insight in potentially disqualifying side effects of Org 214007-0 on glucose metabolism, we have carefully evaluated its effect on glucose metabolism in the liver by a mass isotopomer distribution analysis (MIDA) approach. Prior studies that were performed to gain insight in the effects of prednisolone on glucose metabolism in mice, showed that the MIDA approach was able to specifically quantify the actions of prednisolone on hepatic glucose metabolism [41]. Other tests, such as ipGTT and ipITT, have therefore not been performed with Org 214007-0 as these would have no additional value. MIDA has also successfully been applied to study glucose metabolism in humans [42] and was adapted for use in mice [43]. Using this method it was found that prednisolone administration of 10 mg/kg/day for 7 days significantly reduced glycogen storage in the liver by reducing glucokinase and glycogen synthase fluxes, while a dose of Org 214007-0 that was equi-efficacious in reducing arthritis did not. Surprisingly, hepatic gluconeogenesis was not affected by prednisolone treatment while the commonly accepted idea is that GCs stimulate gluconeogenesis *via* induction of genes like PEPCK and G6Pase. However, induction of these gluconeogenic genes appears to represent an acute effect of GCs. Studies on glucose metabolism after a more chronic GC treatment in man [44], [45], [46] or mice [47] also showed a lack of effect on hepatic gluconeogenesis and rather point at an effect on glucose disposal. Due to limitation of the volume of blood taken during the MIDA experiments plasma insulin levels could not be determined. However, earlier studies have revealed that chronic prednisolone treatment induces a state of reduced hepatic insulin sensitivity and a ‘fasting-like phenotype’ in chow-fed mice [47]. Furthermore, in mice fed with a high fat diet the prednisolone-induced hyperglycemia and hyperinsulinemia was aggravated [41]. So, in contrast to prednisolone, Org 214007-0 did not have any impact on *in vivo* glucose metabolism in the mouse, since no effects on fasting blood glucose levels or on hepatic glucose metabolism were found.

It will be of great interest to further detail effects of Org 214007-0 versus prednisolone for its efficacy in other therapeutic disease models but especially also for its adverse effects, e.g. induction of osteoporosis, diabetes, muscle wasting, and effects on CNS. We consider Org 214007-0 a perfect tool compound to study the pharmacological consequence of dissociating transrepression from transactivation on the therapeutic index of these GCs looking at all of these well known adverse effects. It should be noted, that not for all adverse effects a clear improved therapeutic index can be expected. For instance for bone, it was found that GC-induced osteoporosis in GR-dimer deficient mice is dependent on transrepression rather than transactivation [30].

Thus, Org 214007-0, is a non-steroidal SGRM, with a potentially improved therapeutic index compared to prednisolone. Progress in further preclinical and clinical studies of this new class of SGRMs could lead to a better understanding of the consequences of GC-mediated transrepression and transcativation for the therapeutic index of GCs and to future availability of improved GCs.

## Materials and Methods

### Glucocorticoid receptor binding assay

The glucocorticoid receptor (GR) fluorescence polarisation (FP) binding assay was performed using the commercially available kit from Panvera (Glucocorticoid receptor competitor assay, Green Cat# P2816). (for more detailed information see Supportive Information) (SI text).

### Structural modeling

The binding mode of Org 214007-0 in complex with the GR-LBD was predicted using the flexible docking method Fleksy [48] and images generated using Pymol (The PyMOL Molecular Graphics System, Schrödinger, LLC.).

### Co-factor-peptide recruitment assay

E. coli produced His-GR-LBD (F_602_S mutated) at 10 nM (final concentration) plus 0.032 nM to 10 μM GC compound (final concentration range) were incubated in 20 μL/well in white OptiPlates (in duplo) for 3 hours at 4°C. Hereafter, peptide, representing Transcriptional Intermediary Factor 2 – LXXLL motif 3 (TIF2-3) at 100 nM (final concentration), streptavidin-APC (8 nM, final concentration) and anti-His-Eu (1.25 nM final concentration) were added to a total volume of 50 μL/well and incubation at 4°C was continued for another 20 hours. Fluorescence was measured using Tecan Infinite M1000 and data were analysed by GraphPad Prism.

### Assay in U2OS cells

To determine the repressive activity of GR ligands, cell line U2OS GR.G9, the human osteoblastic cell line U2OS, stably transfected with human GR, was used. Cells (10^4^ cells/well) were incubated in 384-wells plates, in the presence of 50 ng/ml TNFα (R&D systems)/100 ng/ml IFNγ (Peprotech) and compound, for 18 hours. Hereafter, a mixture of two different antibodies to hMCP-1, one Eu-labeled and one APC-labeled, were added. One hour later the time-resolved fluorometric resonance energy transfer (TR-FRET) signal was measured. (for more detailed information see Supportive Information) (SI text).

### Assay in CHO cells

For monitoring both human steroid receptor agonistic as well as antagonistic activity of compounds, Chinese hamster ovary (CHO) K1 cells stably co-transfected with the specific human steroid receptor and its respective reporter construct were used (as previously described) [49].

For human glucocorticoid receptor (GR)-specific activity CHO-GR B4.8 cells containing both recombinant human GR as well as a reporter construct consisting of the mouse mammary tumor virus (MMTV) promotor and the luciferase reporter gene, were used. Compound was incubated alone (in the agonistic setup) or with 50 nM dexamethasone (in the antagonistic setup) overnight. Hereafter, 200 µl medium was removed and 50 µl luciferine substrate solution from the LucLite luminescence kit (Packard, Meriden, USA) was added. After 10 minutes luminescence of each sample was counted and percentage maximal agonistic efficacy was related to the maximal efficacy of prednisolone. Percentage maximal antagonistic efficacy was related to the maximal antagonistic efficacy of reference GR antagonist Org 34116. Similar procedures were followed for the other human steroid receptor assays (for more detailed information see Supportive Information) (SI text).

### Gene expression profiling in HepG2 cells

HepG2-8/97-WS.5 cells, deprived from serum for 18 hours, were stimulated with glucocorticoid and 0.5 mM cAMP for 6 hours. RNA was isolated and used for double stranded cDNA synthesis using the One-Cycle Target Labeling Kit (Affymetrix Santa Clara, CA). This cDNA was used as a template for the preparation of biotin-labeled cRNA using the GeneChip IVT Labeling Kit (Affymetrix Santa Clara, CA). Biotin-labeled cRNA was fragmented and hybridized at 45°C for 16–17 hours to the Human Genome U133A 2.0 Array or the Human Genome U133 Plus 2.0 Array (Affymetrix, Santa Clara, CA). (for more detailed information see Supportive Information) (SI text). Arrays were stained with phycoerythrin-streptavidin conjugate (Molecular Probes, Eugene, OR), and the signals were amplified by staining the array with biotin-labeled anti-streptavidin antibody (Vector Laboratories, Burlingame, CA) followed by phycoerythrin-streptavidin. The arrays were laser scanned with an GeneChip Scanner 3000 6G (Affymetrix, Santa Clara, CA) according to the manufacturer's instructions. Data was saved as raw image file and quantified using GCOS (Affymetrix). Genes with a fold change of >2 and a p-value of <0.05 (after correction for multiple testing) were selected. The mean fold induction by prednisolone of these genes was compared with the mean fold induction of these genes by Org 214007-0 (more detailed information in Supportive Information) (SI text).

### Gene expression profiling in THP-1 cells

THP-1 cells were incubated with glucocorticoid in the presence of either DMSO (control) or IFNγ/TNFα (220 ng/ml/375 ng/ml). After 6 hours total RNA was isolated and further processed for microarray hybridization as described for the HepG2 cells. The data from all microarray experiments have been deposited in the NCBI Gene Expression Omnibus (GEO) under accession number (identifier will be included in proof). A therapeutic index for Org 214007-0 was calculated based on the difference in the ratios of mean fold change in induction of top 25 genes and repression of top 25 genes by Org 214007-0 versus prednisolone (see Figure S2). Expression of FKBP51, DUSP1 and GILZ was confirmed by Q-PCR according to the same procedure as described for the HepG2 cells. In this case THP1 cells were stimulated with 40 ng/ml IFNγ and 60 ng/ml TNFα and a whole dose range of prednisolone and Org 214007-0 was tested. Expression of FKBP51, MCP-1, IL-6 and IL-8 protein was measured by specific AlphaLISAs (PerkingElmer; #AL244C [MCP-1], #AL223C [IL-6] and #AL224C [IL-8]) according to the manufacturer manual. For the FKBP51-specific AlphaLISA anti-FKBP51 Mab (Abnova, Cat H0002289-M01) and Goat anti-IgG FKBP51 polyclonal (Santa Cruz Biotechnology, Cat sc-11514) were used and the same procedure was followed as for the cytokine-specific AlphaLISAs (more detailed information in Supportive Information) (SI text).

### ChIP-Seq analysis

From the THP-1 samples that were used for gene expression analysis after 6 hours, samples were drawn after 1 hour and used for ChIP–Seq analysis at Genpathway (Carlsbad, CA). For precipitation of genomic DNA regions antibody against GR (Santa Cruz sc-8992) was used. Details are provided in SI text. The obtained amplified DNA libraries were sent to Illumina Sequencing Services (San Diego, CA) for sequencing on a Genome Analyzer II. Sequence files were analyzed with the Genomatix Genome Analyzer Workbench from Genomatix (Genomatix Software GmbH, Munich). Sequences were clustered using default parameter settings and aligned against the human genome version GRCh37/hg19. Clusters were merged using the Replicate Analysis module from the Workbench package (more detailed information in SI text).

### Human whole blood LPS- or PMA/anti-CD28-induced cytokine release

Peripheral blood from healthy volunteers was collected into lithium heparinised tubes, diluted with RPMI 1640 medium (without phenol red) (Gibco BRL) and used within 3 hours after collection. Diluted blood (final dilution 5x) was pre-incubated in cell culture 96-wells flat-bottom plates (Nunc) together with compound for 1 hour and further incubated with 1 μg/ml Lipopolysaccharide (E. coli serotype 0111:B4, Sigma) or 100 ng/ml phorbol 12-myristate 13-acetate (PMA, Sigma)/100 ng/ml mouse anti-human CD28 antibody (Sanquin, The Netherlands) for 24 hours in an incubator at 37°C, 6% CO2 and 95% humidity. Hereafter, supernatant was carefully collected and tested at the most optimal dilution (predetermined by a titration study on control samples) in a human TNFα-specific ELISA (for the LPS stimulated samples) or in a human IL-5-specific or human G-CSF-specific ELISA (for the PMA/anti-CD28 stimulated samples) (Duoset, R&D systems USA).

### Animals

All animal procedures and experiments received approval by the Ethics Committee on Animal Experiments of the University Medical Center Groningen or the Ethics Committee on Animals Experiments of MSD Oss and were according to the recognized guidelines. Female Balb/c mice (Charles River, Sulzfeld, Germany), male C57BL/6J OlaHsd (Harlan, Zeist, the Netherlands) and male DBA/1J/BOM mice (Bomholtgard, Ry, Denmark) were group-housed under controlled conditions with a constant temperature (19–21°C), a 12-h light/dark cycle and *ad libitum* access to water and standard laboratory chow pellets. For MIDA analysis, C57Bl6 mice were equipped with a permanent right jugular vein catheter afterwhich mice were allowed to recover.

### Acute inflammation mouse models

For both the LPS-induced TNFα as well as the anti-CD3 induced IL-2 model, female Balb/c mice of 8–12 week old were used. Mice were treated orally with 0.2 ml compound or vehicle (water/mannitol (5%) with 5% DMSO/5% chremophore for the LPS model or with 0.5% gelatine for the anti-CD3 model). One hour after oral treatment animals were challenged i.p. with 0.2 ml of either 20 μg/mouse LPS (Sigma, E. coli serotype 055:B5) or 5 µg/mouse hamster anti-CD3 monoclonal (BD Biosciences Pharmingen, art. nr. 553057, clone 145-2C11). Ninety minutes after LPS injection or 3 hours after anti-CD3 challenge, blood samples were collected and serum levels of TNFα or IL-2 were determined via specific ELISAs (BD Biosciences Pharmingen, San Diego, CA, USA). In the experiment that included treatment with RU486 to block GR, mice were dosed subcutaneously with 50 mg/kg RU486 30 minutes before the oral compound dosing. ED50 and % efficacy (maximal percentage inhibition of TNFα or IL-2 release) are determined using a 3 parameter sigmoidal curve calculation. Statistical analysis of the results was performed using an one-way ANOVA (log) test.

### Mouse CIA model

The experiment was essentially performed as described previously [50]. At day 0 mice were immunized at the base of the tail with 100 μg bovine type II collagen (UMC Nijmegen, The Netherlands) in Complete Freund's Adjuvant (CFA, Difco) enriched with 2 mg/ml *Mycobacterium tuberculosis* H37RA (Difco). At day 21 mice were boosted by an i.p. injection of 100 μg bovine type II collagen dissolved in saline. After disease onset, mice with an arthritis score ranging from 0.25 to 1.25 were divided into matched groups (n = 12). Animals were orally treated once daily with vehicle (0.5% gelatin/5% mannitol in water) or compound for 21 to 23 days. The clinical severity of arthritis (arthritis score) was graded and scored as described [51]. To assess the effects of treatments, the area under the curve (AUC) of arthritis score (subtracting baseline AUC of arthritis score on day 0) was used. One day after the final treatment animals were sacrificed and the knee and ankle joints were imaged using a Faxitron X-ray, Modl MX-20 digital imaging system and analysed using Specimen (Version 2.0.1). The bone destruction was scored on a scale of 0–5 as described previously [51]. The cumulative scores of 2 joints (right ankle and knee) were used as radiological scores. From H&H-stained sections, articular cartilage destruction and inflammatory infiltrate in the right knee joint were scored on a scale of 0–3. The whole study was carried out in a blinded fashion. Statistical analysis was performed using one-way ANOVA.

### Micro array on muscle tissue mRNA from mice

Two and a half hours after the final treatment animals were sacrificed and samples were collected from the thigh muscle (*musculus tensor fasciae latae* and *musculus rectus femoris*). Samples were stored immediately at −80°C and send to Covance (Princeton, New Jersey) for RNA extraction and subsequent analysis of gene expression on the Mouse Genome 430A 2.0 array. For statistical analysis, the. CEL files obtained after hybridization were analyzed with the R (www.r-project.org) and the BioConductor software package (www.bioconductor.org) as described previously [36]. Normalization was done using gcrma. Building of the experimental design and calculation of the ratios was done with the limma package. Differentially expressed probe sets were selected on basis of the fold change and the adjusted p-value (Benjamini-Hochberg correction). Multivariate data analysis and clustering was done with standard methods in the R software package (www.r-project.org). A therapeutic index for Org 214007-0 was calculated in the same way as was described for the THP-1 microarray data.

### Mouse fasting glucose and MIDA model for liver glucose metabolism

For fasting blood glucose levels, male C57Bl/6J OlaHSD mice (n = 8 per group, ∼25 g) were treated daily by oral gavage for 28 days with either prednisolone (0.5 or 10 mg/kg/day), an equipotent dose of Org 214007-0 (0.17 or 1.5 mg/kg/day), vehicle (0.5% gelatin/5% mannitol in water) or no treatment as a stress control. Eighteen hours after the last oral treatment and 9 hours after start of starvation, blood samples were taken from the tail vein and directly used for glucose measurements using “One touch Ultra” glucose meter from Lifescan EuroFlash (Lifescan Benelux Beerse, Belgium). The mouse MIDA study was performed as described previously by van Dijk et al. [43], [52]. Briefly, individually housed C57Bl/6J OlaHSD mice (n = 8) were treated orally with prednisolone (10 mg/kg/day), Org 214007-0 (1.5 mg/kg/day) or vehicle (0.5% gelatin/5% mannitol in water) daily for 7 days. On the third day, mice were cannulated in the right jugular vein. After 7 days of treatment, mice were placed in small Plexiglas cages, which allowed collection of blood samples frequently in freely moving mice. Twenty four hours after the final dose and after a 9 hours starving period, a tail vein blood sample was used for a glucose measurement after which mice were shortly anesthetized to attach the infusion lines before the experiment. Filter paper was placed under the wired floor cages to collect urine samples and replaced hourly. Mice received an infusion of a sterilized aqueous solution containing [U-^13^C]glucose (13 μmol/ml), [2-^13^C]glycerol (160 μmol/ml), [1-^2^H]galactose (33 μmol/ml) and paracetamol (1 mg/ml) at a rate of 0.54 ml/hr for 6 hours. During the experiment blood glucose was measured and blood spots for GCMS measurements were collected from tail tips just before the start of the infusion and at hourly intervals. Blood spots and urine filter papers were air-dried and stored at room temperature until analysis. Glucose and paracetamol-glucuronic acid (Par-GLcUa) were extracted from blood spot and urine filter papers, respectively, derivatised, and measured by GC-MS, essentially as described previously [43], [52]. The fractional isotopomer distribution according to GC-MS was corrected for fractional distribution due to the natural abundance of ^13^C by multiple linear regression to obtain the excess mole fraction of mass isotopomers M_0_-M_6_ due to incorporation of infused labeled carbohydrates. From isotope dilution and infusion rates, the flux rates of the following metabolic pathways could be calculated: 1) *de novo* synthesis of G6P, 2) glucokinase, 3) glucose-6-phosphatase, 4) glycogenolysis, and 5) glycogen synthesis. The method of calculation was performed as described before [43], [52], [53].

## Supporting Information

Figure S1 **Org 214007-0 partially induces expression of GC-regulated genes in the human HepG2 cell line.** HepG2 cells were incubated with either vehicle, Org 214007-0 or prednisolone for 6 hours. RNA was isolated and expression of tyrosine aminotransferase (TAT) and glucose 6-phosphatase (G6Pase) was quantified by Q-PCR, expressed as fold induction in comparison to vehicle treated cells.
(TIF)

Figure S2 **Calculation of the relative therapeutic index (TI) based on micro array data.** For the two sets of top 25 genes either induced or repressed with the highest change fold by prednisolone and Org 214007-0 in THP1 cells (shown in Figure 4A and 4B respectively), a mean fold change (FC) was calculated (shown on the Y-axis as a 2log ratio compared to control, the expression of the genes without compound). MeanFC_I_ is the mean fold change of expression of genes induced by prednisolone (Pred) or Org 214007-0 (Org). MeanFC_R_ is the mean fold change of expression of genes repressed by prednisolone (Pred) or Org 214007-0 (Org). The ratio between induction and repression for both compounds can be calculated as meanFC_I_ – meanFC_R_. For Org 214007-0 this is 1.21–0.71 = 0.50. For prednisolone this is 3.61–1.76 = 1.84. The difference is thus 1.84–0.50 = 1.34. This corresponds to a mean difference in fold change of 2?1.34 = 2.53 fold change, the relative therapeutic index (TI) of Org 214007-0. The percentage maximal efficacy of induction of genes and repression of genes by Org 214007-0, in comparison to prednisolone (both set at 100%), in this setting becomes respectively: 2?1.21/2?3.61 * 100% = 18.9% and 2?0.71/2?1.76 * 100% = 48.3%.
(TIF)

Figure S3 **Org 214007-0 behaves as a partial agonist in THP1 cells.** In comparison to prednisolone, Org 214007-0 behaves as a partial agonist in THP1 cells with a stronger partiality on induction of genes (FKBP51, DUSP1 and GILZ) than on the repression of genes (MCP-1, IL-8 and IL-6) under inflammatory conditions. Induction of FKBP51, DUSP1 and GILZ were evaluated by Q-PCR on mRNA isolated from THP1 cells, incubated for 6 hours with compound. Repression of MCP-1, IL-8 and IL-6 was measured on THP1 cell supernatant collected 6 hours after incubation with compound and stimulus.
(TIF)

Figure S4 **The anti-inflammatory effect of Org 214007-0 is mediated through the glucocorticoid receptor.** Mice were treated p.o. either with vehicle, Org 214007-0 (0.5, 1, 2 or 4 mg/kg) in a co-treatment schedule with either a vehicle or RU486 (50 mg/kg) s.c. injection. TNFα was quantified 1.5 h after LPS challenge. Data is represented as mean ± SEM. An one way ANOVA was used for statistical analysis. An asterix (*) represents significant difference from vehicle (p<0.05).
(TIF)

Figure S5 **Org 214007-0 reduces all parameters scored by histological examination of the knee joints of CIA mice.** At the end of the CIA study (after 3 weeks of daily oral treatment with equipotent dosages of prednisolone and Org 214007-0) knee joints were fixed in 4%-buffered formaldehyde and decalcified. After decalcification the joints were washed, dehydrated and embedded in paraffin. Serial sections of at least 7 μm were stained by Hematoxylin-Eosin (for cellular infiltration and exudate formation into the joint space and bone apposition), or by toluindin blue staining (for proteoglycan depletion and cartilage and/or bone destruction of the joint). Org 214007-0, at 0.5 and 1.5 mg/kg, significantly reduced the formation of new bone (bone apposition), the infiltration of inflammatory cells in the joint cavity (exudate) or joint tissue (infiltration), cartilage destruction and proteoglycan depletion in the articular cartilage (PG depletion). Each of the histological parameters is scored at a scale of 0–3. Mean score of each group of mice (n = 12) is indicated (± SEM). **  =  significantly different from vehicle (p<0.01; ANOVA-test).
(TIF)

Figure S6 **Org 214007-0, at a dose that is equi-efficacious to prednisolone, induces less fasting glucose.** Mice (n = 8 per group) were treated p.o., once daily, for 7 days with either vehicle (V), 10 mg/kg prednisolone (P) or 1.5 mg/kg Org 214007-0 (O). Blood glucose levels (mean ± SEM) were measured at day 8, after 9 hours of fasting. ** p<0.01: significantly different (Student's t-test) from vehicle-treated group; # p<0.05: significantly different (Student's t-test) from prednisolone-treated group.
(TIF)

Text S1 More detailed information on Material and Methods and Table S1, Table S2, Table S3 and Table S4.
(DOC)
