# Supplementary figures and images for: Org 214007-0: A Novel Non-Steroidal Selective Glucocorticoid Receptor Modulator with Full Anti-Inflammatory Properties and Improved Therapeutic Index
Source: PLoS One. 2012 Nov 12;7(11):e48385. doi: 10.1371/journal.pone.0048385 (PMC3495945; doi:10.1371/journal.pone.0048385)

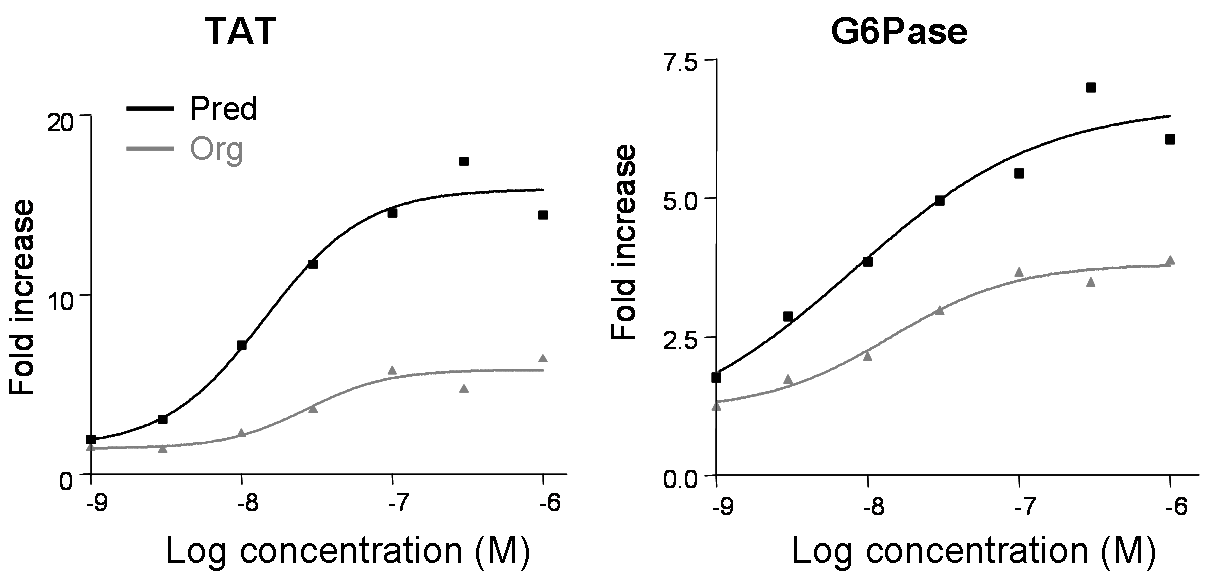

Supplement: Figure S1 — Org 214007-0 partially induces expression of GC-regulated genes in the human HepG2 cell line. HepG2 cells were incubated with either vehicle, Org 214007-0 or prednisolone for 6 hours. RNA was isolated and expression of tyrosine aminotransferase (TAT) and glucose 6-phosphatase (G6Pase) was quantified by Q-PCR, expressed as fold induction in comparison to vehicle treated cells. (TIF) [file pone.0048385.s001.tif]

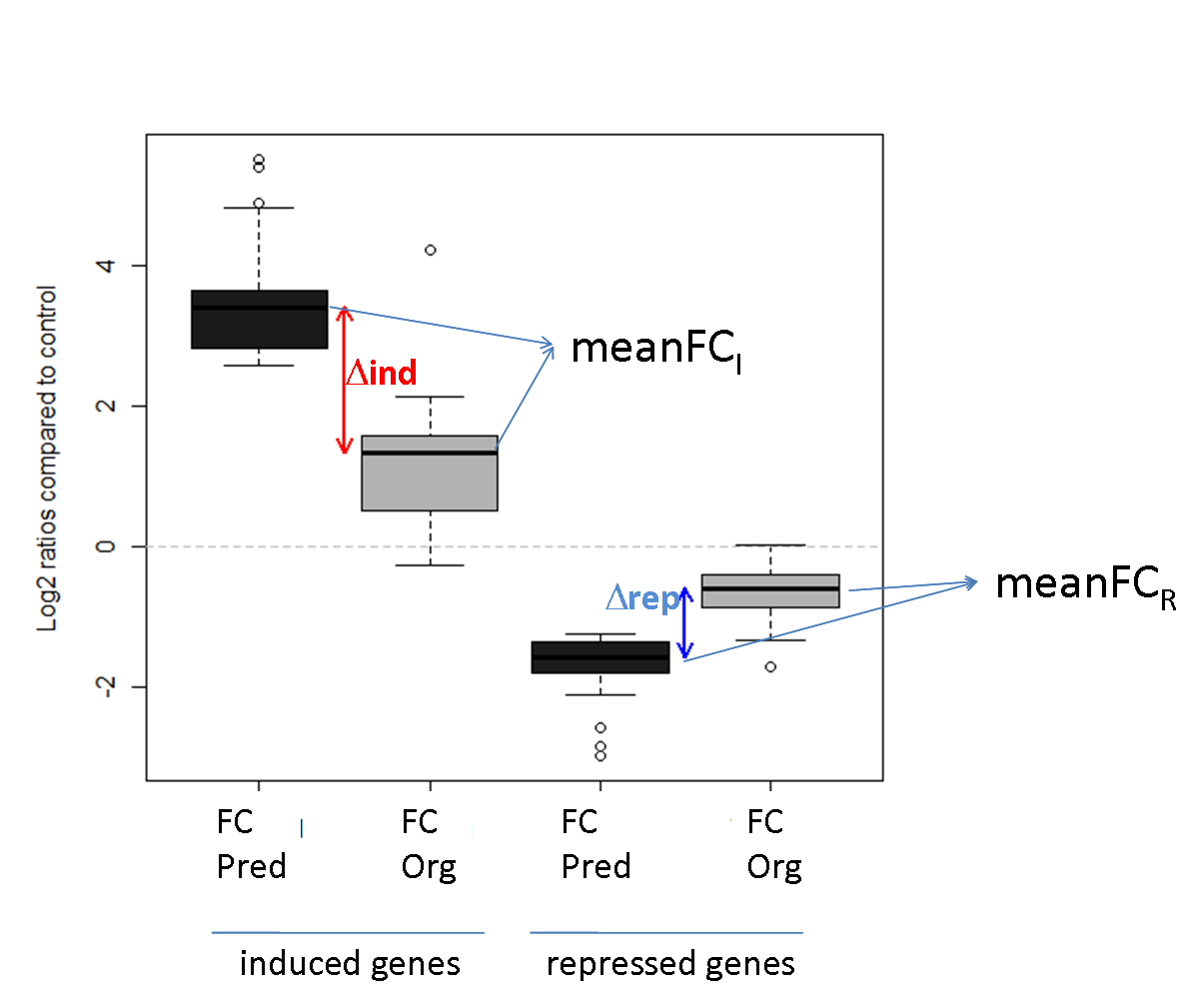

Supplement: Figure S2 — Calculation of the relative therapeutic index (TI) based on micro array data. For the two sets of top 25 genes either induced or repressed with the highest change fold by prednisolone and Org 214007-0 in THP1 cells (shown in Figure 4A and 4B respectively), a mean fold change (FC) was calculated (shown on the Y-axis as a 2log ratio compared to control, the expression of the genes without compound). MeanFCI is the mean fold change of expression of genes induced by prednisolone (Pred) or Org 214007-0 (Org). MeanFCR is the mean fold change of expression of genes repressed by prednisolone (Pred) or Org 214007-0 (Org). The ratio between induction and repression for both compounds can be calculated as meanFCI – meanFCR. For Org 214007-0 this is 1.21–0.71 = 0.50. For prednisolone this is 3.61–1.76 = 1.84. The difference is thus 1.84–0.50 = 1.34. This corresponds to a mean difference in fold change of 2?1.34 = 2.53 fold change, the relative therapeutic index (TI) of Org 214007-0. The percentage maximal efficacy of induction of genes and repression of genes by Org 214007-0, in comparison to prednisolone (both set at 100%), in this setting becomes respectively: 2?1.21/2?3.61 * 100% = 18.9% and 2?0.71/2?1.76 * 100% = 48.3%. (TIF) [file pone.0048385.s002.tif]

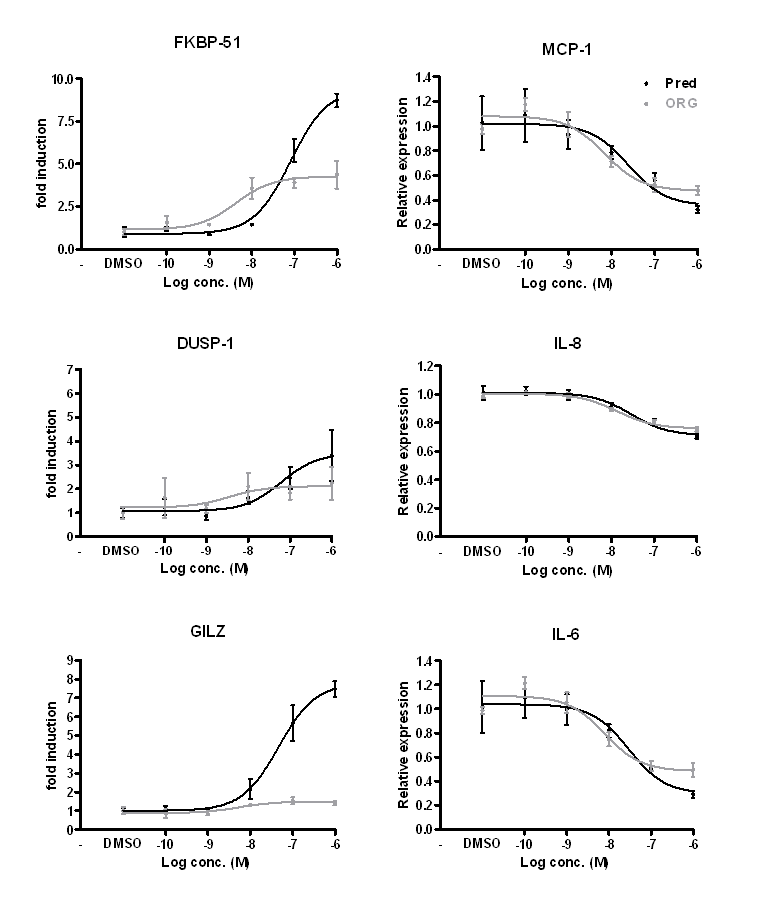

Supplement: Figure S3 — Org 214007-0 behaves as a partial agonist in THP1 cells. In comparison to prednisolone, Org 214007-0 behaves as a partial agonist in THP1 cells with a stronger partiality on induction of genes (FKBP51, DUSP1 and GILZ) than on the repression of genes (MCP-1, IL-8 and IL-6) under inflammatory conditions. Induction of FKBP51, DUSP1 and GILZ were evaluated by Q-PCR on mRNA isolated from THP1 cells, incubated for 6 hours with compound. Repression of MCP-1, IL-8 and IL-6 was measured on THP1 cell supernatant collected 6 hours after incubation with compound and stimulus. (TIF) [file pone.0048385.s003.tif]

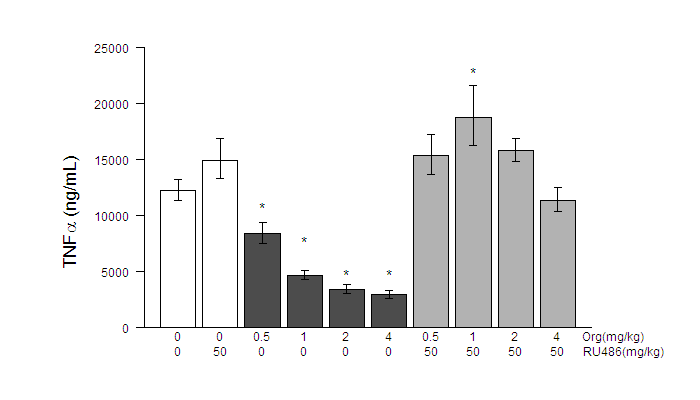

Supplement: Figure S4 — The anti-inflammatory effect of Org 214007-0 is mediated through the glucocorticoid receptor. Mice were treated p.o. either with vehicle, Org 214007-0 (0.5, 1, 2 or 4 mg/kg) in a co-treatment schedule with either a vehicle or RU486 (50 mg/kg) s.c. injection. TNFα was quantified 1.5 h after LPS challenge. Data is represented as mean ± SEM. An one way ANOVA was used for statistical analysis. An asterix (*) represents significant difference from vehicle (p<0.05). (TIF) [file pone.0048385.s004.tif]

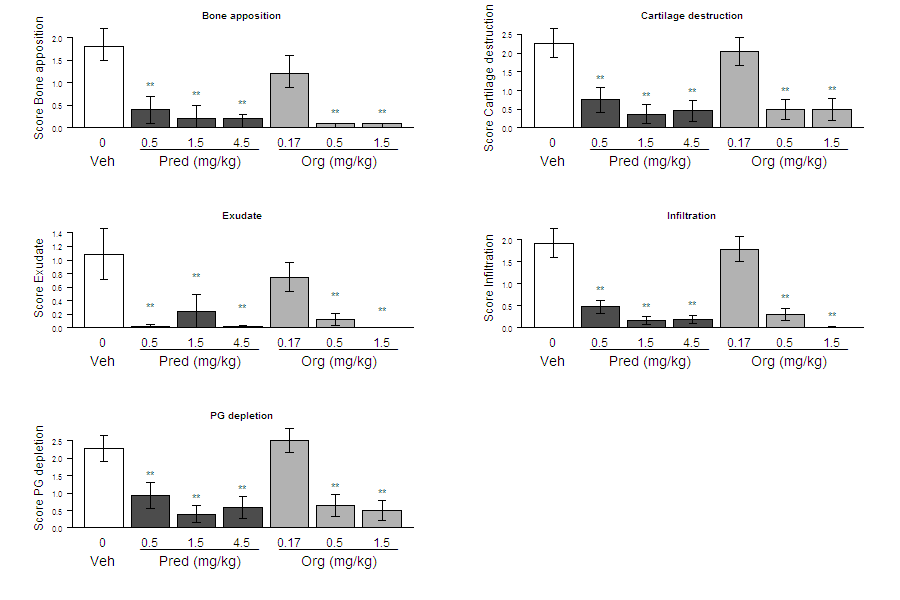

Supplement: Figure S5 — Org 214007-0 reduces all parameters scored by histological examination of the knee joints of CIA mice. At the end of the CIA study (after 3 weeks of daily oral treatment with equipotent dosages of prednisolone and Org 214007-0) knee joints were fixed in 4%-buffered formaldehyde and decalcified. After decalcification the joints were washed, dehydrated and embedded in paraffin. Serial sections of at least 7 μm were stained by Hematoxylin-Eosin (for cellular infiltration and exudate formation into the joint space and bone apposition), or by toluindin blue staining (for proteoglycan depletion and cartilage and/or bone destruction of the joint). Org 214007-0, at 0.5 and 1.5 mg/kg, significantly reduced the formation of new bone (bone apposition), the infiltration of inflammatory cells in the joint cavity (exudate) or joint tissue (infiltration), cartilage destruction and proteoglycan depletion in the articular cartilage (PG depletion). Each of the histological parameters is scored at a scale of 0–3. Mean score of each group of mice (n = 12) is indicated (± SEM). ** = significantly different from vehicle (p<0.01; ANOVA-test). (TIF) [file pone.0048385.s005.tif]

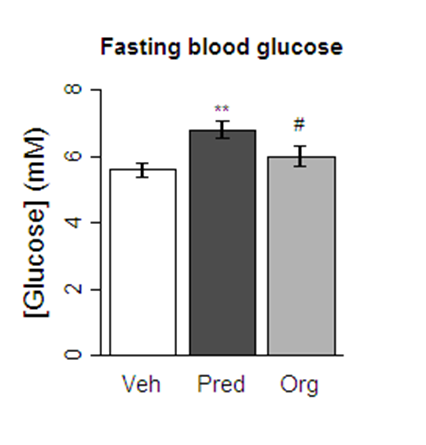

Supplement: Figure S6 — Org 214007-0, at a dose that is equi-efficacious to prednisolone, induces less fasting glucose. Mice (n = 8 per group) were treated p.o., once daily, for 7 days with either vehicle (V), 10 mg/kg prednisolone (P) or 1.5 mg/kg Org 214007-0 (O). Blood glucose levels (mean ± SEM) were measured at day 8, after 9 hours of fasting. ** p<0.01: significantly different (Student's t-test) from vehicle-treated group; # p<0.05: significantly different (Student's t-test) from prednisolone-treated group. (TIF) [file pone.0048385.s006.tif]
