# Supplementary material for: Org 214007-0: A Novel Non-Steroidal Selective Glucocorticoid Receptor Modulator with Full Anti-Inflammatory Properties and Improved Therapeutic Index
Source: PLoS One. 2012 Nov 12;7(11):e48385. doi: 10.1371/journal.pone.0048385 (PMC3495945; doi:10.1371/journal.pone.0048385)
Supplement: Text S1 — More detailed information on Material and Methods and Table S1, Table S2, Table S3 and Table S4. (DOC) [file pone.0048385.s007.doc]

**Supportive information (text)**

**Glucocorticoid receptor binding assay**

The glucocorticoid receptor (GR) fluorescence polarisation (FP) binding assay was performed using the commercially available kit from Panvera (Glucocorticoid receptor competitor assay, Green Cat# P2816). Briefly, recombinant human GR was added to a fluorescent glucocorticoid ligand (Fluormone™ GS1) in the presence of competitor test compounds in black round bottom 384 well microtitre plates for 1.5 to 2.5 hours at roomtemperature. Fluorescence polarization was measured on an Analyst with suitable 485nm excitation and 535nm emission interference filters for FP.

**Assay in U2OS cells**

U2OS GR.G9 cells (10^4^ cells/well) in Dulbecco's MEM / HAM F12 medium supplemented with 5% charcoal treated bovine calf serum (Hyclone) (v/v) and Penicillin (10 U/ml) -Streptomycin (10 μg/ml) were incubated in 384-wells plates, in the presence of 50 ng/ml TNFα (R&D systems) / 100 ng/ml IFNγ (Peprotech) and compound, for 18 hours in a humidified atmosphere at 37°C under 6.5% CO2. Hereafter, a mixture of 2.2 nM anti-hMCP-1 labeled with fluorescent donor Europium (Eu) and 16.6 nM anti-hMCP-1 labeled with fluorescent acceptor Allophycocyanin (APC) was added to each well and left at roomtemperature for 1 hour. Finally, the time-resolved fluorometric resonance energy transfer (TR-FRET) signal was measured with the Victor^2^TM V, 1420 Multilabel Counter (Wallac). Using 4-parameter curve fitting, IC50 values for the compounds were determined. Besides IC50, also percentage maximal efficacy of each compound related to the maximal efficacy of the reference prednisolone was given.

**Assay in CHO cells**

For human glucocorticoid receptor (GR)-specific activity CHO-GR B4.8 cells containing both recombinant human GR as well as a reporter construct consisting of the mouse mammary tumor virus (MMTV) promotor and the luciferase reporter gene, were used. Compound was incubated alone (in the agonistic setup) or with 50 nM dexamethasone (in the antagonistic setup) in microtiter white culture plates seeded with 2.4 x 10^4^ cells / 200 µl Dulbecco’s MEM/Nutrient Mix F12 medium (Gibco) supplemented with 5% charcoal treated defined bovine calf serum supplement (DBCSS, Hyclone, Utah, USA), and 10 U/ml Penicillin-10μg/ml Streptomycin (GibcoBRL) overnight at 37°C in the dark under a humidified atmosphere flushed with 5% CO2 in air. Hereafter, 200 µl medium was removed and 50 µl luciferine substrate solution from the LucLite luminescence kit (Packard, Meriden, USA) was added. After 10 minutes (when cell lysis was complete) luminescence of each sample was counted using a Topcount microplate luminescence counter. Percentage maximal agonistic efficacy was related to the maximal efficacy of dexamethasone. Percentage maximal antagonistic efficacy was related to the maximal antagonistic efficacy of reference GR antagonist Org 34116. EC50 value was calculated by 4-parameter curve fitting using the effect of 6 compound concentrations.

Similar procedures were followed for the other human steroid receptor assays containing combinations of human progesterone receptor B (PR-B) and MMTV-Luc, human androgen receptor (AR) and MMTV-Luc, human chimeric glucocorticoid-mineralocorticoid receptor (GGM, Trapp et al. (1994), *Neuron* 13: 1457) and MMTV-Luc, human estrogen receptor alpha (ERα) and rat oxytocin-Luc (45), and human estrogen receptor beta (ERβ) and rat oxytocin-Luc, respectively.

**McPhail test**

The McPhail (or anti-McPhail) test was used to evaluate potential progestational (or anti-progestational) activity of Org 214007-0 on the endometrium in estrogen-primed immature rabbits after once-daily administration. The female NZW rabbits were housed in pairs in a light- and temperature-controlled room, fed daily with 40 - 50 g standard pelleted food and with free access to tap-water. Before treatment with compounds rabbits were primed daily in the morning with a subcutaneous dose of 2 μg estradiol benzoate for 8 days. In the agonistic McPhail test the rabbits were subsequently treated for 5 days with positive control medroxyprogesterone acetate (0.05 mg/kg/day; 0.25 mg in total) or Org 214007-0 (0.5 mg/kg/day; 2.5 mg/kg in total) (n=2 per treatment group). Autopsy was performed one day after the last treatment. The uterus was dissected and two different parts of each uterine horn were fixed in Bouin's fluid for 12 hours. Transverse sections were prepared, stained with haematoxylin-eosin and evaluated microscopically. Endometrial development was scored on a 0-4 scale as follows: score 0: no development of glands; score 1: start of development of glands; score 2: clear-cut development of glands with ducts beginning to branch; score 3: about 2/3 of stroma bearing glandual tissue; score 4: more than 2/3 of stroma bearing glandular tissue. A score of 2 or higher in both rabbits was evaluated as a positive resonse.

For the anti-McPhail test, the estrogen-primed rabbits were subsequently treated for 7 days with inducer progesterone (0.25 mg/kg/day s.c.) in combination with an oral treatment of either placebo, reference RU486 (4 mg/kg/day) or Org 214007-0 (0.5 mg/kg/day). The uterus preparation and evaluation of the endometrium was performed as described above.

**Gene expression profiling in HepG2 cells**

HepG2-8/97-WS.5 cells were seeded at 2.10^5^ cells/mL in a 24-well plate in DMEM/F12 medium (Gibco), supplemented with 80 U/mL penicillin, 80 μg/mL streptomycin and 5% Fetal Bovine Serum (Invitrogen). The following day, cells were washed once with PBS and medium was replaced by serum-free medium for 18 hours. Hereafter, cells were stimulated with glucocorticoid and 0.5 mM cAMP for 6 hours. RNA isolation was carried out by TRIZOL (Invitrogen) treatment. To enhance the A260/A230 ratio, samples were cleaned using the RNeasy mini kit (Qiagen) with an additional DNase step on column to ensure DNA-free RNA. RNA quantity and quality were determined using the Agilent Nanodrop bioanalyzer. For all samples subjected to micro-array hybridization, the RIN (RNA integrity number) was 9.0 – 10. Double-stranded cDNA was synthesized from 1.5 μg total RNA using the One-Cycle Target Labeling Kit (Affymetrix Santa Clara, CA), and used as a template for the preparation of biotin-labeled cRNA using the GeneChip IVT Labeling Kit (Affymetrix Santa Clara, CA). Biotin-labeled cRNA was fragmented at 1 μg/μl following the manufacturer’s protocol. After fragmentation, cRNA (10μg) was hybridized at 45°C for 16-17 hours to the Human Genome U133A 2.0 Array or the Human Genome U133 Plus 2.0 Array (Affymetrix, Santa Clara, CA). Following hybridization, the arrays were washed, stained with phycoerythrin-streptavidin conjugate (Molecular Probes, Eugene, OR), and the signals were amplified by staining the array with biotin-labeled anti-streptavidin antibody (Vector Laboratories, Burlingame, CA) followed by phycoerythrin-streptavidin. The arrays were laser scanned with an GeneChip Scanner 3000 6G (Affymetrix, Santa Clara, CA) according to the manufacturer's instructions. Data was saved as raw image file and quantified using GCOS (Affymetrix).

For Q-PCR, cDNA was synthesized by the use of 1 μg RNA that was added to 1 μg of random primer (Promega) in a total volume of 15 μL, heated to 70 ºC for 10 min and cooled on ice. M-MLV reverse transcriptase (200 units) (Promega), 10 nmol of each dNTP and RT buffer was added to a total volume of 25 μL and incubated for 10 min at 22 ºC, 50 min at 55 ºC and 15 min at 75 ºC. cDNA samples were stored at -20 ºC. For Q-PCR, cDNA was diluted 125 times in milliQ and 5 μL was added to 12.5 μL of SYBR green mix (Applied Biosystems cat. No. 4309155) , 2.5 μL of each primer (~20 ng/μl) and 2.5 μL of milliQ. The following primer-pairs were used: for GAPDH: 5’-CCACATCGCTCAGACACCAT-3’ and 5’-CCAGGCGCCCAATACG-3’; for PEPCK (of PCK1): 5’-TCCCATTGAAGGCATTATCTTTG-3’ and 5’-GCCAGCTGAGAGCTTCATAGACT-3’; for TAT: 5’-TACAGACCCTGAAGTTACCCAG-3’ and 5’-TAAGAAGCAATCTCCTCCCGA-3’; for G6Pase (of: G6PC?): 5’-TGCTGCTCAAGGGACTG-3’ and 5’-GAATGGGAGCCACTTGCTG-3’. Quantitative PCR was performed on an ABI7900HT real time thermo cycler (Applied Biosystems) and results were analyzed by SDS2.1 software.

**Gene expression profiling in THP-1 cells**

THP-1 cells (10^6^ cells/ml) were incubated with a single high dose (1 μM) of either prednisolone or Org 214007-0 in the presence of either DMSO (control) or IFNγ/TNFα (220ng/ml / 375 ng/ml). After 6 hours (predetermined optimal time-point for maximal induction of most GR-regulated genes), total RNA was isolated and further processed for microarray hybridization as described for the HepG2 cells. For a selection of the GC-regulated genes the expression profile was confirmed. In these experiments THP1 cells were stimulated with 40 ng/ml IFNγ and 60 ng/ml TNFα and a whole dose range of prednisolone and Org 214007-0 was tested. Expression of FKBP51, DUSP1 and GILZ was measured by Q-PCR according to the same procedure as described for the HepG2 cells. In this case the following primer-pairs were used: for GAPDH: 5’-CCACATCGCTCAGACACCAT-3’ and 5’-CCAGGCGCCCAATACG-3’; for FKBP51: 5’-AAAAGGCCAAGGAGCACAAC-3’ and 5’-TTGAGGAGGGGCCGAGTTC-3’; for DUSP1: 5’-GTACATCAAGTCCATCTGAC-3’ and 5’-GGTTCTTCTAGGAGTAGACA-3’; for GILZ: 5’-AGTGCCTCCGGAGCCAGC-3’ and 5’-ATGATTCTTCACCAGATCC-3’. For the evaluation of protein expression, cells were incubated overnight with a whole dose range of compounds. Supernatants were collected to determine cytokine levels and cell lysates were generated by repeated freeze-thaw cycles to measure expression of FKBP51 protein. Expression of FKBP51, MCP-1, IL-6 and IL-8 was measured by specific AlphaLISAs (PerkingElmer; #AL244C [MCP-1], #AL223C [IL-6] and #AL224C [IL-8]) according to the manufacturer manual. Briefly, 5 μL of sample was added to 20 μL of a mix (freshly prepared) AlphaLISA Anti-Analyte Acceptor beads (10 μg/mL final) and Biotinylated Antibody Anti-Analyte (1 nM final) into a 384-well white OptiPlate-384. Samples were incubated for 60 min at 23˚C. StreptAvidin-Donor beads (40 μg/mL final) were added and incubated for another 30 min at 23˚C in the dark. Plates were measured using an EnVision-Alpha Reader. For the anti-FKBP51 AlphaLISA, customized FKBP51-specific Mab (Abnova, Cat H0002289-M01)-coated beads as Acceptor beads and biotinylated Goat anti-IgG FKBP51 polyclonal (Santa Cruz Biotechnology, Cat sc-11514) as Biotinylated Antibody Anti-Analyte were used.

**ChIP-SEQ analysis**

Cells were fixed with 1% formaldehyde for 15 min and quenched with 0.125 M glycine. Chromatin was isolated by adding lysis buffer, followed by disruption with a Dounce homogenizer (cells) or motor pestle (tissue). Lysates were sonicated and the DNA sheared to an average length of 300-500 bp. Genomic DNA (Input) was prepared by treating aliquots of chromatin with RNase, proteinase K and heat for de-crosslinking, followed by ethanol precipitation. Pellets were resuspended and the resulting DNA was quantified on a NanoDrop spectrophotometer. Extrapolation to the original chromatin volume allowed quantitation of the total chromatin yield.

An aliquot of chromatin (28.5 ug) was precleared with protein A agarose beads (Invitrogen). Genomic DNA regions of interest were isolated using 4 ug antibody against GR (Santa Cruz sc-8992). Complexes were washed, eluted from the beads with SDS buffer, and subjected to RNase and proteinase K treatment. Crosslinks were reversed by incubation overnight at 65 C, and ChIP DNA was purified by phenol-chloroform extraction and ethanol precipitation.

Quantitative PCR (QPCR) reactions were carried out in triplicate on specific genomic regions using SYBR Green Supermix (Bio-Rad). The resulting signals were normalized for primer efficiency by carrying out QPCR for each primer pair using Input DNA. ChIP and Input DNAs were prepared for amplification by converting overhangs into phosphorylated blunt ends and adding an adenine to the 3’ ends. Illumina adaptors were added and the library was size-selected (175-225 bp) on an agarose gel. The adaptor-ligated libraries were amplified for 18 cycles. The resulting amplified DNAs were purified, quantified, and tested by QPCR at the same specific genomic regions as the original ChIP DNA to assess quality of the amplification reactions. Amplified DNAs (DNA libraries) were sent to Illumina Sequencing Services for sequencing on a Genome Analyzer II. Sequence files were analyzed with the Genomatix Genome Analyzer Workbench from Genomatix (Genomatix Software GmbH, Munich). Sequences were clustered using default parameter settings and aligned against the human genome version GRCh37/hg19. Clusters were merged using the Replicate Analysis module from the Workbench package. In short, this results in a file with chromosomal coordinates with new merged clusters for each position in which a cluster is found for one or more conditions. For each of these merged clusters, the reads and normalized enrichment for each of the conditions is given. Additionally, for each merged clusters P-values for each pairwise comparison of the three conditions were also calculated.

**Table S1. Activity of Org 214007-0 for the other human steroid receptors, stably co-transfected with their respective reporter readouts in CHO cells.**

| **Steroid receptor** | **% agonist**  **potency** | **EC50 (nM)** | **%**  **efficacy** | **n** | **% antagonist**  **potency** | **IC50 (nM)** | **%**  **efficacy** | **n** |
| --- | --- | --- | --- | --- | --- | --- | --- | --- |
| PR (B) | <0.03 | >100 | na | 4 | 0.46 (±0.2) | 19.4 (±7.6) | > 87 | 5 |
| MR | <0.1 | >1000 | na | 2 | <1.27 | >1260 | > 40 | 2 |
| AR | <0.13 | >100 | na | 4 | <3.83 | >1000 | na | 5 |
| ERα | <0.01 | >100 | na | 5 | <1.86 | >1000 | na | 2 |
| ERß | <0.01 | >100 | na | 5 | <0.9 | >1000 | na | 5 |

The data are expressed as the mean (±SD) of the relative agonistic or antagonistic activity as compared to the respective reference compounds. na = not applicable; n = number of experiments

**Table S2. In vivo progestagenic and anti-progestagenic activity of Org 214007-0 in female rabbits, using the agonistic and antagonistic McPhail test.**

| **Agonistic test** | | **Antagonistic test** ***(antagonizing 0.025 mg/kg/day progesterone treatment)*** | |
| --- | --- | --- | --- |
| **treatment (mg/kg/day)** | **score** | **treatment (mg/kg/day)** | **score** |
| MPA (0.05) | 2.5 | placebo | 3.0 |
| Org 214007-0 (0.5) | 0.5 | RU486 (4) | 0.5 |
|  |  | Org 214007-0 (0.5) | 3.31 |

For both the agonistic and antagonistic test immature female NZW rabbits (n=2 per treatment group) were primed with estrogen. For the agonistic test the rabbits were subsequently treated for 5 days with Org 214007-0 or the positive control medroxyprogesterone acetate (MPA). One day after the last treatment the uterus was dissected and endometrial development was scored on a 0-4 scale. A score value >2 is a positive response. Org 214007-0 showed no progestational activity (score 0.5).

For the antagonistic test, after oestrogen priming, rabbits were treated for 7 days with progesterone and placebo, reference RU486 or Org 214007-0. One day after the last treatment the uterus was dissected and endometrial development was scored on a 0-4 scale. A significant reduction of the progesterone-induced endometrial transformation was observed with RU486 (score 0.5). After treatment with Org 214007-0 no reduction of the endometrial transformation was observed (score 3.31), indicative for no anti-progestagenic activity

**Table S3. Repressive activity of 10 μM Org 214007-0 in comparison to 10 μM prednisolone on cytokine release of human whole blood cells stimulated with LPS for 24 hours**

|  | **TNFα** | **n** | **IL-1β** | **n** | **IL-6** | **n** | **IL-8** | **n** |
| --- | --- | --- | --- | --- | --- | --- | --- | --- |
| Prednisolone | 100 | 16 | 100 | 3 | 100 | 3 | 100 | 3 |
| ORG 214007-0 | 81 (± 7) | 12 | 64 (± 8) | 3 | 54 (± 6) | 3 | 49 (± 55) | 3 |

The data are expressed as the mean percentage inhibition (±SD) by Org 214007-0 compared to that by prednisolone (set at 100%); n = number of experiments

**Table S4. Pharmacokinetic parameters of Org 214007-0 and prednisolone in mice.**

|  | **Org 214007-0** | | | **Prednisolone** | | |
| --- | --- | --- | --- | --- | --- | --- |
|  | | 4 μmol/kg i.v. | 10 μmol/kg p.o. | | 10 μmol/kg i.v. | 20 μmol/kg p.o. |
| Tmax (h) | |  | 0.5 | |  | 0.3 |
| Cmax (nmol/ml) | | 2.19 | 2.12 | | 18 | 11.5 |
| T1/2 eli (h) | | 6.4 | 5.3 | | 0.26 | 0.4 |
| AUClast (h*nmol/ml) | | 10.1 | 23.2 | | 7.1 | 11 |
| AUCinf (h*nmol/ml) | | 10.8 | 24.2 | | 7.1 | 11 |
| Vz (L/kg) | | 3.4 | 3.2 | | 0.5 | 1.2 |
| Cl (L/h/kg) | | 0.4 | 0.4 | | 1.4 | 1.8 |
| MRTinf (h) | | 7.9 | 7.1 | | 0.4 | 0.8 |
| Vss (L/kg) | | 2.9 |  | | 0.5 |  |
| F (% bioavailability) | |  | 89.7 | |  | 77.4 |

For each time-point 3 female Balb/c mice are used. Tmax = Time of maximum observed concentration (in case of i.v. administration Tmax = 0 h); time is expressed in hours (h). Cmax = concentration corresponding to Tmax and expressed in nmol/ml, for i.v. dosing Cmax is extrapolated to T=0. T 1/2 eli(mination) (terminal half-life) = ln(2)/λ_z_ expressed in h(ours). AUClast = Area under the curve from the time of dosing (Dosing-time) to the last measurable concentration. AUCinf = predicted Area Under the Curve from the time of dosing extrapolated to infinity and expressed in “h*nmol/ml”. Vz = Volume of distribution based on the terminal phase = Dose / λ_z_* AUCinf and expressed inL/kg. Cl = total body clearance = Dose / AUCinf and expressed in L/h/kg. MRTinf = mean residence time (MRT) extrapolated to infinity for non-infusion models, expressed in h. Vss = gives an estimate of the volume of distribution at steady-state and is expressed in L/kg. F = % bioavailability; assuming linear kinetics, the % bioavailability after oral administration is calculated by dividing the AUCinf obtained after oral administration by the mean expected normalized AUCinf after i.v. administration of that dose (x 100%) (AUCiv = 100% by definition). The bioavailability is expressed in %. i.v. = intraveneously, p.o. = per oral
